# Developing Lactic Acid Bacteria as an Oral Healthy Food

**DOI:** 10.3390/life11040268

**Published:** 2021-03-24

**Authors:** Wei-Kuang Lai, Ying-Chen Lu, Chun-Ren Hsieh, Chien-Kei Wei, Yi-Hong Tsai, Fang-Rong Chang, You Chan

**Affiliations:** 1 Graduate Institute of Natural Products, Kaohsiung Medical University, College of Pharmacy, Kaohsiung 807, Taiwan; u101831003@kmu.edu.tw (W.-K.L.); u97831003@kmu.edu.tw (C.-K.W.); r960134@kmu.edu.tw (Y.-H.T.); 2 Department of Food Science, National Chiayi University, Chiayi City 600, Taiwan; biolyc@mail.ncyu.edu.tw (Y.-C.L.); s1060505@mail.ncyu.edu.tw (C.-R.H.); 3 Drug Development and Value Creation Research Center, Kaohsiung Medical University, Kaohsiung 80708, Taiwan; 4 Department of Medical Research, Kaohsiung Medical University Hospital, Kaohsiung Medical University, Kaohsiung 80708, Taiwan; 5 Department of Marine Biotechnology and Resources, National Sun Yat-sen University, Kaohsiung 80424, Taiwan; 6 Institute of Biochemistry, Microbiology, and Immunity, Chung Shan Medical University, Taichung City 40201, Taiwan

**Keywords:** lactic acid bacteria (LAB), oral pathogen, caries, periodontal disease, *Streptococcus mutans*, *Porphyromonas gingivalis*, *Lacticaseibacillusparacasei*

## Abstract

Lactic acid bacteria have functions in immunoregulation, antagonism, and pathogen inhibition. The purpose of this study was to evaluate the effectiveness of lactic acid bacteria (LAB) in countering oral pathogens and develop related products. After a series of assays to 450 LAB strains, 8 heat-inactivated strains showed a strong inhibitory effect on a caries pathogen, *Streptococcus mutans*, and 308 heat-inactivated LAB strains showed a strong inhibitory effect on a periodontal pathogen, *Porphyromonas gingivalis*. The key reasons for inhibiting oral pathogens were bacteriocins produced by LAB and the coaggregation effect of the inactivated cells. We selected *Lacticaseibacillus* (*Lb*) *paracasei* 111 and *Lb.* *paracasei* 141, which had the strongest inhibitory effects on the above pathogens, was the main oral health food source. The optimal cultural conditions of *Lb. paracasei* 111 and *Lb. paracasei* 141 were studied. An oral tablet with a shelf life of 446 days made of the above strains was developed. A 40 volunteers’ clinical study (CSMUH IRB number: CS05065) was conducted with this tablet in the Periodontological Department of the Stomatology Research Center, Affiliated Hospital of Chung Shan Medical University (Taiwan). After 8 weeks of testing, 95% and 78.9% of patients showed an effect on reducing periodontal pathogens and improving probing pocket depth, respectively, in the oral tablet group.

## 1. Introduction

Lactic acid bacteria (LAB) constitute a broad heterogeneous group of generally food-grade microorganisms. Recent research showed the genus *Lactobacillus* could be classified into 25 genera [1]. LAB naturally present in fermented foods and is involved in the food process, used as a starter of fermentation to improve aroma, digestibility, flavor, and shorten ripening periods. In addition to these important effects, a series of enzymatic reactions are involved in producing metabolites such as acetaldehyde, hydrogen peroxide, lactic acid, and peptides during the fermentation of food [2,3]. Fermented milk products, such as cheese and yogurt, were consumed by humans about 8000–10,000 years ago. The discovery and characterization of LAB changed the views of food fermentation [4].

In the past twenty years, most LAB have been used as probiotics in foods, and it is well-known that probiotics have several beneficial health effects in humans. They play an important role in the protection of the hosts against harmful microorganisms, as well as regulating the immune system [5,6,7,8]. Recently, studies showed that probiotics were effective for some conditions of humans. However, in some conditions, buying probiotics is like money being thrown into the toilet [9]. Probiotics had a short-term and individualized impact on the mucosal community structure and intestinal transcriptome. When antibiotics were used, it would enhance the colonization of probiotics in the intestinal mucosa. The benefits of taking probiotics may be offset by the recovery of the intestinal mucosa [10]. The studies highlighted the need to develop individualized probiotics in the protection of intestinal mucosa.”

Bacteriocins are simple ribosomally biosynthesized peptides with an antibacterial activity that are easily degraded by proteolytic enzymes. LAB bacteriocins are heat-resistant, active in a wide pH range, and furthermore, are colorless, tasteless, and odorless, but will not exist in the human body for a long time [11].

LAB could inhibit the growth of oral caries pathogens. A previous study showed that *Lacticaseibacillus* (*Lb*) *paracasei* [12] from healthy teeth of volunteers in Thailand was isolated and showed good antibacterial ability against many oral pathogenic bacteria, such as *Porphyromonas gingivalis, Streptococcus mutans, and S. salivarius *[13]. *Levilactobacillus brevis* BBE-Y52 could adhere to oral epithelial cells. Coincubation of this strain with *S. mutans* ATCC 25175 increased the IL-10 to IL-12p70 ratio in peripheral blood mononuclear cells, which indicated that *Lb. brevis* BBE-Y52 could alleviate inflammation and may confer benefits to the health of the host by modulating the immune system [14]. Periodontitis-causing bacteria, such as *Fusobacterium nucleatum* and *P. gingivalis,* can degrade amino acids into volatile sulfur compounds and cause halitosis. *Weisella cibaria* could effectively co-aggregate with *F. nucleatum* and inhibited its proliferation to prevent halitosis [15].

Orthogonal array tables were used in many research experiments. They are based on a system of tabulated designs that enable the maximum number of variables to be estimated in a balanced manner with merely a few numbers of experiments and to enhance the efficiency and reproducibility of experiments [16]. The Arrhenius equation could describe the relationship between product quality change rate and storage temperature. It could be used to shorten the time of testing of product storability and product shelf life [17].

Previous studies showed a concentration of *S. mutans* associated with a high risk of developing tooth decay was cfu/mL > 10^5^ [18]. They also showed a single copy gene-based real-time PCR method was used to test the concentration of *P. gingivalis* in the saliva of periodontal disease patients. The median values of *P. gingivalis* in pathogen-positive subjects varied between 1.61 × 10^3^ to 3.57 × 10^4^ genomes/mL of saliva among healthy subjects and between 7.09 × 10^3^ to 1.26 × 10^6^ genomes/mL of saliva among subjects with periodontitis [19]. Hence, in the confirmation test, we took the pathogen concentration as 2.0 × 10^7^ cfu/mL.

The purpose of this study was to screenings of inactive LAB cells, and a platform for evaluating the effectiveness of LAB in countering oral pathogens, such as *S. mutans* and *P. gingivalis* was constructed. A series of tests of methods and a clinical trial were carried out.

## 2. Materials and Methods

### 2.1. Anti-Carious and Anti-Periodontal Pathogens Functional Tests

#### 2.1.1. Lactic Acid Bacteria

A total of 450 strains of LAB were deposited in the laboratory of Professor Ying-Chen Lu in National Chiayi University (Taiwan), including *Lactobacillus acidophilus*, *Lacticaseibacillus* (*Lb*.) *brevis*, *Lactobacillus delbrueckii* subsp. *bulgaricus*, *Lb. casei*, *Limosilactobacillus fermentum*, *Lactobacillus gasseri*, *Lactobacillus helveticus*, *Lactobacillus delbrueckii* subsp. *lactis*, *Lb. paracasei*, *Lactiplantibacillus plantarum*, *Limosilactobacillus reuteri*, *Lb. rhamnosus*, *Ligilactobacillus *(*Llb*.),* salivarius*, *Fructobacillus fructosus*, *Streptococcus thermophilus*, *Bifidobacterium lactis*, which are shown in Table S1. All LAB strains were cultured in de Man, Rogosa and Sharpe (MRS) broth (Difco 288130, Becton, Dickinson and Company, Franklin Lakes, NJ, USA) at 37 °C for 18 h and stored at −80 °C in 10% glycerol [20].

#### 2.1.2. Bacteria of Dental Caries and Periodontal Disease

*S. mutans* (BCRC 16002) was purchased from the BCRC, Food Industry Development Institute (Taiwan), while *P. gingivalis* ATCC 33277 was purchased from American Type Culture Collection (ATCC), USA. *S. mutans* strains was cultured in BHI agar (Brain Heart Infusion agar, Merck, KGaA, Darmstadt, Germany) at 37 °C with 5% CO_2_ for 3 days and stored at 5 °C. *P. gingivalis* strains was cultured in Brucella blood agar plates (BBAP, 2.81% (*w/v*) Brucella broth base (Sigma-Aldrich, St. Louis, Missouri, USA), 5.0% (*v/v*) sheep blood (C. M. P., R.O.C.), 1.8% agar (*w/v*)) at 37 °C with 5% CO_2_ for 3 days and stored at 5 °C [21].

### 2.2. Screening of LAB Inhibition Oral Pathogens

#### 2.2.1. Crude Bacteriocin Extraction

LAB were cultured in liquid MRS medium at 37 °C for 18 h, and inactivated at 75 °C for 30 min and then cooled down to 25 °C. 30 mL of culturing fluid was centrifuged at 16,000× *g* for 10 min, and the supernatant was taken. The supernatant was added 16.83 g of 80% saturated ammonium sulfate water solution (56.1%, *w/w*) and centrifuged at 8000× *g* for 20 min to precipitate the crude bacteriocins. After the supernatant was removed, the precipitated material was redissolved with 1.5 mL of pure water to obtain the crude bacteriocin extract [22].

#### 2.2.2. Anti-Oral Pathogenic Bacteria Test

The antimicrobial assay was carried out using the Agar well diffusion method [23]. *S. mutans* (1.0 × 10^9^ cfu/mL) was cultured in liquid BHI medium for 3 days. The BHI agar plate was spread evenly with *S. mutans* (1.0 × 10^7^ cfu/mL) and punched to make a 7.32 mm diameter well. One hundred microliter crude bacteriocin extraction samples were placed into the wells and cultured at 37 °C with 5% CO_2_ for 72 h. The diameter of inhibition zones was then measured, the experiment was repeated in triplicate. The assay for *P. gingivalis* was conducted in a similar way, but the agar medium was changed to BBAP.

### 2.3. Confirm Test of Anti-Cariogenic Bacteria and Anti-Periodontal Disease Bacteria

#### 2.3.1. Testing of Coincubation of LAB and Oral Pathogens

The target strains with the strongest inhibitory effect on different oral pathogens were selected as the test group, and 3 strains selected randomly from 450 LAB strains were assigned as the control group. Moreover, they were cultured in liquid MRS medium at 37 °C for 18 h. The cultured LAB fluids were inactivated at 75 °C for 30 min, and adjusted to 2.0 × 10^7^ cells/mL, 2.0 × 10^8^ cells/mL, and 2.0 × 10^9^ cells/mL, respectively. Each 1 mL of LAB fluid was mixed with 1 mL 2.0 × 10^7^ cfu/mL *S. mutans* and coincubation in 8 mL BHI liquid medium at 37 °C for 0, 6, and 24 h. Then 1 mL of the above mixture was cultured in a BHI agar medium at 37 °C with 5% CO_2_ for 72 h. Using a similar method, the testing of coincubation of LAB and *P. gingivalis* was conducted, but the agar medium was changed to BBAP. These tests were performed in triplicate. After coincubation, the concentrations of pathogens were checked at every time point of coincubation by a plate count method [24].

#### 2.3.2. Observing of Co-Aggregation

*S. mutans* was cultured in BHI agar at 37 °C with 5% CO_2_ for 3 days. *P. gingivalis* was cultured in BBAP at 37 °C with 5% CO_2_ for 3 days. Bacterial cells from agar plates were harvested, and the suspension was made in sterile phosphate-buffered saline (PBS). The final optical density was 1 (about 1.0 × 10^9^ cfu/mL) and measured by a spectrophotometer at 600 nm (Eppendorf, Hamburg, Germany). Then, the LAB suspensions were inactive at 75 °C for 30 min. Further, the LAB suspensions were inactivated at 75 °C for 30 min. Equal volumes (1 mL) of LAB suspensions and pathogen suspensions were divided into test tubes and mixed by vortexing. Control tubes contained 2 mL of a suspension of each bacterial species. Absorbance was measured at 5 min. The percentage of coaggregation was determined as following Equation (1):

[(Ax + Ay)/2 − A(x + y)]/[(Ax + Ay)/2] × 100(1)

where A represented absorbance, x and y represented each of two suspensions in the control tubes, and (x + y) represented their mixture. Then, coaggregation was monitored by microscopy at 400 times magnification after Gram staining [20].

### 2.4. The Study of Optimal Culture Conditions of Target Strains

The study of optimal culture conditions of target strains was conducted in two experiments. The first experiment was using L_18_ (2^1^ × 3^7^) orthogonal table [25] to configure seven factors (medium (skim milk powder + glucose), growth factor, culture temperature, controlled pH, starter amount (percentage at 2.0 × 10^8^ cfu/mL of target strain), feed speed, and the culturing time) with a wider range of each level value. In the second experiment, four important significant factors were selected from the results of the first experiment; the range of each level value was reduced and configured with the L_9_ (3^4^) orthogonal table [16] for more precise experiments (Tables S2–S7). These tests were performed in triplicate.

### 2.5. Preparation, Drying the Cultured Fluid of Target Strains, and Identification of the Strains

#### 2.5.1. Preparation, Drying the Cultured Fluid Samples of the Target Strains

The cultured samples of the target strains from the optimum culturing conditions were inactivated at 75 °C for 30 min and freeze-dried with freeze-dryer (LyoDryer serial no. 08-650 model Nolyodryer ST5B, Kingston, NY, USA). The processes were as follows: the cultured fluid was frozen to −20 °C, and the water was vacuumed and dried under 0.1 tor. At this time, the temperature continued to drop to −40 °C. Since then, the temperature was increased with a gradient of 1.2 °C/60 min. The final temperature was 35 °C and kept for 60 min. In this way, the target strain solid samples with moisture below 3.5% were obtained [26].

#### 2.5.2. Identification of Strains

The aforementioned LAB strain solid samples were outsourced (Genomics BioSci & Tech. Co., Ltd., New Taipei City, Taiwan) to identify bacterial species and conducted random amplification of polymorphic DNA (RAPD) analysis.

### 2.6. Healthy Food Product Design, Manufacturing, and Quality Evaluation

#### 2.6.1. Shape, Size, and Prescription Design

In considering that the oral product lasts for a time period in the mouth to achieve an anti-oral pathogen effect, the product was designed as a tablet type. The diameter was 13 mm, the thickness was 7 mm, and the weight was 1000 mg of the round tablet. The main materials were derived from inactive cell powders of the target strains, which were decided according to a confirmed test. Other raw materials were flavors, maltodextrin, oligosaccharide, silicon dioxide, and xylitol, of which usage and dosage were formed in accordance with the standard of food additives published by the Food and Drug Administration, Ministry of Health and Welfare, Executive Yuan of the Republic of China. The preloaded pressure of the tablet was 1100 kg/cm^2^, and the main pressure of the tablet was 5000 kg/cm^2^. The package was a composite material, with polyethylene terephthalate (PET) on the top and aluminum foil on the bottom.

#### 2.6.2. Measurement of Heat-Inactivated LAB Biomass

The LAB solids obtained by freeze-drying and other processing methods were pulverized. Ten grams of powder were dissolved in 90 g of pure water, and its starch and protein were treated with 1.0% of amylase (Amylase DS, Amano Enzyme USA. Co., Ltd., Elgin, IL, USA) and 1.0% of protease (PROTEASE P “AMANO” 6, Amano Enzyme USA. Co., Ltd., Elgin, IL, USA) at 40 °C for 2 h. The numbers of LAB cells were counted with a 400× microscope in a counting chamber.

### 2.7. Quality Evaluation and Improvement of Tablets

Antipathogen activity, disintegration time, flavor, hardness, and loss on drying were used as the index for quality evaluation. In the antipathogen activity, 10 g of tablets were dissolved into 90 mL of sterile water, and 1 mL of the above solution and 1 mL of *S. mutans* (2.0 × 10^7^ cells/mL) in BHI medium was mixed and coincubated at 37 °C for two minutes. The mixture was cultured in BHI agar with 5.0% of CO_2_ for 3 days, and the number of colonies of *S. mutans* was counted, with *P. gingivalis* being treated in a similar way with a BBAP [19]. The specification of antipathogen activity (counts of oral pathogenic bacteria after coincubation) was ≤ 20 colonies. The disintegration time was measured by disintegration analyzer (CT-2, Shinko Seiki Industries Co., Ltd., Kobe-shi, Hyogo, Japan), and the specification was 510 ± 90 s. A 9-score hedonic sensory flavor test was used to evaluate tablets by 10 well-trained panelists, where score 9 meant extremely liked, 8 meant liked very much, 7 meant liked, 6 meant liked a little, 5 meant not liked nor disliked, 4 meant disliked a little, 3 meant disliked, 2 meant disliked very much, and 1 meant disliked extremely. The specification was the mean of hedonic score ≥ 7.0; hardness was tested by a AEF-20 hardness tester (Digitech Co., Ltd., Osaka, Japan); and the specification was ≥ 21.0 kg. The loss on drying was measured with a MX-50 moisture analyzer (A&D Company, Limited, Tokyo, Japan), and the specification was ≤ 5.0% on 110 °C. If any index tested was outside the specifications, the design would be modified until the index met the specifications.

### 2.8. The Storability Test and Setting the Product Shelf Life

The test index was the same as the index of quality evaluation. The Arrhenius equation was used as a tool to shorten the test time [17] and was described as follows: k = k_o_ exp (−E_a_/R)/T. k was the rate of quality change, k_o_ was the exponential coefficient, E_a_ was the activation energy, R was the constant, and T was the absolute temperature. The logarithm was taken on both sides to obtain the following formula in the equation: Log_10_ k = log_10_ k_o_ + (−E_a_/R)/T, log_10_ k_o_ was the intercept, and -E_a_/R was the slope. Therefore, taking Log_10_ k as the vertical coordinate and 1/T as the abscissa, the linear equation was obtained. Tablets were stored at 15 °C for 60 days, 25 °C for 30 days, 35 °C for 15 days, and 45 °C for 7.5 days. The quality index was determined, the quality change rate was calculated, and the regression equation between the quality change rate and temperature to estimate product storability was found. In addition, the quality change period from tablet production to the lower limit of the specifications could be predicted by the Arrhenius equation, and this period was set as the shelf life of products.

### 2.9. Clinical Trial of Periodontal Therapy

This clinical trial was a randomized, double-blind, placebo-controlled study conducted at the Department of Periodontology of the Stomatology Research Center of the Chung Shan Medical University Hospital (Taiwan). Forty patients (mean age 47.7 ± 9.8 years) who had not previously received any type of periodontal therapy were enrolled. Bleeding on probing, probing depth, and clinical attachment level measurements were assessed by the same examiner throughout the whole course. All participants gave written consents, and Chung Shan Medical University Hospital obtained ethical approval for this study (CSMUH No.: CS05065). The volunteers were randomized into oral tablet product and placebo groups. Each volunteer of the group received three tablets containing target strains or none per day for a consecutive 8 weeks; one tablet within thirty minutes after each meal. Clinical measurements, including oral flora total counts, periodontal pathogen counts (*P. gingivalis, P. intermedia),* and improvement of periodontal pocket depth and halitosis test were measured at the beginning of the test, 4 weeks, and 8 weeks after enrollment [13].

#### 2.9.1. The Growth Inhibition of Periodontal Pathogens

For inhibiting the growth of periodontal pathogens, plaque samples were collected and processed. Subgingival plaque samples were collected with two points from the periodontal pocket of the lesion site and then placed in 1 mL of reduced transport fluid [27]. The samples were transferred immediately to an anaerobic environment, where they were vortex-mixed and homogenized. Part of each sample was serially diluted and plated in BBAP at 37 °C with 5% CO_2_ for 48–96 h. The total bacterial counts were indicated by their morphology under dark-field microscopy, long-wave UV light fluorescence test (Blak-Ray Lamp, model UVL-56, long-wave UV- 366 nm, Fisher Scientific, Pittsburgh, PA, USA), and the colonies with different morphology were Gram-stained. *P. gingivalis* was shown as a white colony under UV light, while *P. intermedia* was shown as a black colony with lacerate morphology under UV light [28].

#### 2.9.2. Improvement of Periodontal Probing Depth *(*PPD)

This measure was made with specially marked periodontal probes held parallel to the tooth and inserted under the free gingival margin, and gently “walked” to the base of the sulcus (i.e., pocket). The blunt periodontal probes (P2N, HU-FRIEDY) were typically marked with rings or bands that measured distance in millimeters. The mean PPD across three teeth of 1 patient formed the periodontal probing depth as an estimate, and the mean PPD < 5 mm was defined as disease improvement, with PPD being generally measured as the distance from the base of the sulcus to the top of the free gingival margin [29].

#### 2.9.3. Changes of Halitosis Following Treatment

To measure the degree of volatile sulfur compounds (VSC) in the mouth, objects were requested to receive measurement by a halimeter (Breathron, BT-814, Yoshida, Tokyo, Japan) for two to three hours after breakfast. Improvement of halitosis was the ratio of baseline and 4th week ≥ 1.1, and the ratio of baseline and 8th week ≥ 1.1 [30].

### 2.10. Statistical Analysis

The SPSS version 25 (IBM Corp, Armonk, NY, USA) software was used to conduct the analyses. One-factor ANOVA was used to determine whether different LAB presented different inhibition zones in Table S1, while the three-factor (different LAB strains, same LAB strain, but at different concentrations, and different incubation times) ANOVA was used to determine whether the counts of oral pathogens after coincubation with LAB were different (Table 1, Table 2 and Table 3). Fisher’s exact test was used to determine whether different groups of periodontal diseases (positive and negative) showed differences in inhibition of periodontal pathogens in volunteers’ oral cavities, whether different groups (placebo group and product group) had differences on probing pocket depth, and whether different groups (placebo group and product group) showed differences on the improvement of halitosis. The McNemar’s test was used to determine whether there was a difference between baseline and 4 weeks and whether there was a difference between baseline and 8 weeks on inhibition of periodontal pathogens in oral cavity measures of the volunteers.

## 3. Results

### 3.1. Screening of LAB Inhibition Pathogens

Four hundred fifty strains of LAB were screened, and the results are shown in Table S1. The inhibitory effect of LAB on *S. mutans* was significantly different, *p* < 0.01, and was the same as the inhibitory effect of LAB on *P. gingivalis*. There were 8 LAB strains showing the inhibitory effects on *S. mutans*, and their mean ± SE (standard error) of the inhibition zone for *S. mutans* was 12.69 ± 0.89 mm. The best one was *Lb. paracasei* 111 with inhibition zone 14.09 ± 0.17 mm (mean ± SD). There were 308 LAB strains showing the inhibitory effects on *P. gingivalis*, and their mean ± SE of the inhibition zone for *P. gingivalis* was 15.73 ± 3.65 mm. The best one was *Lb. paracasei* 141 with inhibition zone 32.56 ± 0.84 mm (mean ± SD). *Lb. paracasei* 111 and *Lb. paracasei* 141 were called “the target strains”.

### 3.2. Results of Confirm Tests of Antipathogens Activity

#### 3.2.1. Testing of Coincubation of LAB with Oral Pathogens

The target strains *Lb. paracasei* 111, *Lb. paracasei* 141 and randomly selected strains *Lb. paracasei* 127, *Llb. salivarius* 285, and *Llb. salivarius* 296 from 450 LAB (control group) were used to confirm the inhibitory effect on *S. mutans* and *P. gingivalis*. The bacterial counts of *S. mutans* and *P. gingivalis* after coincubation with 5 heat-inactivated test strains for a period are shown in Table 1. The inhibitory effect of some test strains, concentrations, coincubation times on *S. mutans* was significant, *p* < 0.01. In Table 1, target strains showed an inhibitory effect on oral pathogens at doses of 10^7^ cells/mL and 10^8^ cells/mL at 0 h. For the longer coincubation time at 6 and 24 h, more residual numbers of pathogenic strains were found. It showed that the effective reaction time started in a few seconds. The target strains showed a strong inhibitory effect on oral pathogens at doses of 10^9^ cells/mL within 24 h. We cited the concentration of 10^9^ cells/mL of target strains for tablet design.

Since the experiment could not be achieved at the real 0 h, another experiment using the same strains of LAB with the same concentrations was devised to control the coincubation time within two minutes, and these results are shown in Table 2. The test strains showed the effect on inhibiting oral pathogens, which were similar to those shown at 0 h in Table 1.

#### 3.2.2. Observing Co-Aggregation

Co-aggregates were produced within 60 s after mixing the heat-inactivated test strains and oral pathogens, and the results were shown in the Table 3 and Figure S1. For *S. mutans*, the strongest coaggregation object was *Lb. paracasei* 111, the moderate coaggregation object was *Llb. salivarius* 296, the slight coaggregation objects were *Lb. paracasei* 127, *Lb. paracasei* 141, *Llb. salivarius* 285. For *P. gingivalis*, the strongest coaggregation object was *Lb. paracasei* 141; the strong coaggregation objects were *Lb. paracasei* 285 and *Lb. paracasei* 296, the moderate coaggregation object was *Lb. paracasei* 127, and *Lb. paracasei* 111 showed a slight coaggregation effect.

Due to *Lb. paracasei* 111 having a good inhibitory effect on *S. mutans* and *Lb. paracasei* 141 having a good inhibitory effect on *P. gingivalis*, these two strains were selected for oral health food development in this study. Moreover, after observing the coaggregation values of *Lb. paracasei* 111 and *Lb. paracasei* 141, the data were under 8.0% within two minutes. It indicated two target strains would not interrupt each other.

### 3.3. The results of the Optimal Culture Conditions Research of Target Strains

After two orthogonal tables (L_18_(2^1^ × 3^7^) and L_9_(3^4^)) experiments (Tables S2–S7) were performed, the optimal cultural conditions for *Lb. paracasei* 111 and *Lb. paracasei* 141 are shown in Table 4.

Consequently, one liter each of *Lb. paracasei* 111 and *Lb. paracasei* 141 were produced under their optimum conditions three times for validation, deriving LAB counts as 4.42 ± 0.52 × 10^9^ cfu/mL and 4.15 ± 0.54 × 10^9^ cfu/mL, respectively.

### 3.4. Results of LAB Powder Preparation and Drying

**Figure 1:**
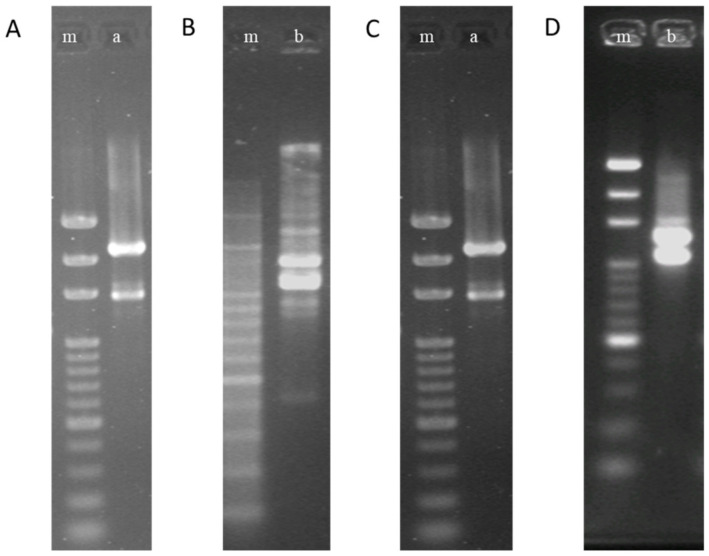
The random amplification of polymorphic DNA (RAPD) patterns of *Lb. paracasei* 111 and *Lb. paracasei* 141 before and after being processed. (**A**,**B**), before processing; (**C**,**D**), after processing. “m” is a marker, “a” is *Lb. paracasei* 111, and “b” is *Lb. paracasei* 141.

Three liters of cultures of *Lb. paracasei* 111 and *Lb. paracasei* 141 were inactivated and freeze-dried. The amounts, loss on drying, and bacterial biomass of *Lb. paracasei* 111 and *Lb. paracasei* 141 are shown in Table 5. The RAPD of the above dried materials were identified, and it was judged that there were no differences from the original strains, as shown in Figure 1.

### 3.5. Results of Tablet Preparation and Quality Evaluation

After several modifications, the composition of the tablets was obtained, as shown in Table 6. Two thousand tablets were manufactured for further research. Tablets were examined to get the values, as shown in Table 7.

### 3.6. Results of Storability Test and Setting the Product Shelf Life

Tablets were stored at experimental conditions, and the quality index and quality change rate were determined to estimate the storability of tablets. A shelf life of at least 446 days was obtained. The indexes, the inhibitory effect as 17 ± 3 (S. mutants counts) and 19 ± 5 (*P. gingivalis* counts), and the flavor quality as 7.2 ± 0.2 (9-point hedonic score), hardness as 21.8 kg, disintegration time as 410 ± 8 s, were determined after 446 days tracked. It was appropriate to set the shelf life to 446 days (Table S8).

### 3.7. Clinical Trials of Tablet Product

#### 3.7.1. Inhibiting the Growth of Periodontal Pathogens

In Table 8, there was no significant change in the placebo group. In the final visit, the inter-group comparison showed statistical significance between product and placebo groups in terms of total bacterial counts in the oral cavity. Further analysis demonstrated that 95% of patients showed no pathogenic growth in the subgingival plaque samples after a 4-week designed tablet treatment. This effectiveness lasted to the final visit compared to the baseline visit (*p* < 0.05). In contrast, there was no significant change in the negative culture rate of oral periodontal pathogens from subgingival plaque samples. This information showed tablets had a significant antimicrobial activity on periodontal pathogens. Compared to the placebo group, the antimicrobial activity of tablets also showed statistical significance between groups at 4-week and 8-week visits (*p* < 0.01).

#### 3.7.2. Improvement of Probing Pocket Depth (PPD)

In Table 9, the PPD was less than 5 mm that was significant in the clinical measures of periodontal disease progression. After taking the target strain tablet for 8 weeks, the improvement of PPD was significant compared to the placebo group (*p* < 0.05).

#### 3.7.3. Changes of halitosis following treatment

In Table 10, the ratio of the comparison of halitosis score between baseline and 4th week (or 8th week) greater than 1.1 was defined as an improvement. After taking the target strain tablet for 4 weeks, the improvement of halitosis score was significant compared to placebo. Around 55% of patients showed improvement of oral halitosis after 4-week and 8 week treatments in the treatment group.

## 4. Discussion

Previous studies showed the antibacterial ability of LAB came from undissociated lactic acid, hydrogen peroxide, bacteriocins, boosting host immunity, and nutrition competition, etc. [3,31,32]. In our study, the pH of cultured media for tablets was adjusted to 5.8, and the undissociated lactic acid was reduced. For pathogenic bacteria hiding deep in caries dens or gums, there are too few immune cells to boost the immunity of hosts as those in the intestine. Hydrogen peroxide would volatilize even under the process of freeze-drying in high vacuum conditions (≤0.1 tors). As for nutrition competition among microbes, only surviving bacteria have the ability; however, the target strains were inactivated by heating; therefore, the nonvolatile and heat-resistant bacteriocins of the target strains and coaggregation characteristic of target strains with other bacteria were the mechanism to inhibit oral pathogens. Heated *Lb. paracasei* 111 showed a 62.3 ± 2.5% co-agglutination effect on *S. mutans* and heated *Lb. paracasei* 141 showed 59.3 ± 0.9% co-agglutination effect on *P. gingivalis* (Table 4). In a previous study, 3 heated *W. cibaria* strains were shown to have 77.2 ± 0.9%, 77.8 ± 2.0% and 55.4 ± 0.7% co-agglutination value on *F. nucleatum* [33]. In our study, when volunteers keep the tablet in their mouth, and tablet may dissolve in the saliva and co-aggregate with oral pathogens. When volunteers gargle, swallow saliva or drink water, the pathogenic bacteria in a co-aggregating form may be vomited or swallowed. It means the tablet is suitable to develop as a monthly cleaner. What is the possible mechanism of the coaggregation effect of LAB on oral pathogens? The most likely reasons were the surface charge, hydrophobic effect, self-polymerization ability and the production of adhesin of LAB [34].

Our tablet showed an inhibitory effect on one of the key cariogenic pathogens, *S. mutans* (Table 2 and Table 4). A previous study showed 245 seven-year-old children consumed chewable tablets containing heat-killing LAB, and the incidence of dental caries was significantly reduced by 42% in treatment for two years [35]. In our study, 40 adult volunteers that had not previously received any type of periodontal therapy were equally divided into two groups: the oral tablet product group and the placebo group. In the product group, 19 volunteers had a PPD index greater than 5 mm. After they consumed tablets for 8 weeks, only 4 volunteers had a PPD index greater than 5 mm (Table 10). A previous study showed 30 volunteers (patients) were divided into two groups examining according to the above method. In the product group, 15 volunteers had a PPD index greater than 5 mm. After they were treated with *Lb. reuteri* tablets for 12 weeks, 11 volunteers had a PPD index greater than 5 mm [36].

However, oral lactobacilli are considered the second most cariogenic bacteria of oral flora, with the genus *Lactobacillus* representing 0.1% of the total salivary flora. Some site-specific studies have also suggested that *Lactobacillus* was the major oral flora of carious lesion, and it is directly associated with deep caries and tooth decay [37]. However, the tablet in our experiments was made of inactivated LAB, which did not have the characteristics of alive LAB in the oral cavity. In addition, it might have bacteriocins of LAB as well as possessing a coaggregation effect to fight pathogens and prevent oral diseases.

## 5. Conclusions

We concluded that bacteriocins and the coaggregating capacity of inactivated LAB to oral pathogens might play key roles in inhibiting oral pathogens. The combination tablets of strains *Lb. paracasei* 111 and *Lb. paracasei* 141 was evidenced by a clinical trial and a series of experiments. This study accomplished the purposes, which described complete methods in developing and producing LAB tablets against dental caries and periodontal pathogens. The research was starting from a systematic screening, finding and confirming the biofunction of LAB, optimizing culture conditions of target strains, processing, formulation, quality evaluation, clinical trials, storability testing, and the shelf life setting of the product. The processes and results can serve as an important model for the research and development of LAB products.

## Figures and Tables

**Table 1:** Counts (mean ± SD) of *S. mutans* and *P. gingivalis* after coincubation with 5 test strains.

Strains of LAB ^a^	Conc. of LAB (cells/mL) ^b^	*S. mutants*			*P. gingivalis*		
		0 h ^c^	6 h	24 h	0 h	6 h	24 h
*Lb. paracasei* 111	1.0 × 10^7^	10.0 ± 1.0	165.0 ± 39.7	172.3 ± 7.0	1050.0 ± 79.4	2356.7 ± 197.3	2693.3 ± 86.2
	1.0 × 10^8^	14.3 ± 3.1	13.3 ± 2.1	23.3 ± 1.5	833.3 ± 32.1	1990.0 ± 65.6	2240.0 ± 81.9
	1.0 × 10^9^	6.7 ± 3.2	6.3 ± 2.5	2.0 ± 2.6	770.0 ± 55.7	1213.3 ± 110.2	1546.7 ± 107.9
*Lb. paracasei* 127	1.0 × 10^7^	84.0 ± 8.2	766.7 ± 41.6	786.7 ± 40.4	86.7 ± 19.3	261.3 ± 28.0	783.3 ± 20.8
	1.0 × 10^8^	75.3 ± 17.7	133.0 ± 17.6	273.3 ± 20.4	78.0 ± 8.0	104.0 ± 25.1	250.7 ± 11.0
	1.0 × 10^9^	58.0 ± 4.6	71.0 ± 7.0	44.0 ± 17.1	73.0 ± 6.1	70.0 ± 22.5	61.0 ± 3.5
*Lb. paracasei* 141	1.0 × 10^7^	1023.3 ± 37.9	2540.0 ± 247.6	2706.7 ± 102.1	18.3 ± 8.6	780.0 ± 75.5	2090.0 ± 50.0
	1.0 × 10^8^	933.3 ± 73.7	2073.3 ± 58.6	2390.0 ± 148.0	13.3 ± 5.7	206.7 ± 25.0	341.0 ± 60.7
	1.0 × 10^9^	793.3 ± 45.1	1073.3 ± 47.3	1360.0 ± 40.0	21.0 ± 2.6	17.7 ± 4.9	37.7 ± 2.1
*Llb. salivarius* 285	1.0 × 10^7^	970.0 ± 50.0	2280.0 ± 141.8	2700.0 ± 78.1	25.0 ± 7.0	893.3 ± 56.9	2306.7 ± 70.2
	1.0 × 10^8^	863.3 ± 15.3	2063.3 ± 35.1	2413.3 ± 174.7	21.0 ± 2.6	177.3 ± 8.6	328.0 ± 44.7
	1.0 × 10^9^	766.7 ± 32.1	950.0 ± 26.5	1310.0 ± 105.4	17.3 ± 1.5	65.7 ± 13.7	49.7 ± 13.4
*Llb. salivarius* 296	1.0 × 10^7^	866.7 ± 25.2	1810.0 ± 127.7	2060.0 ± 81.9	863.3 ± 46.2	1456.7 ± 455.4	2116.7 ± 196.6
	1.0 × 10^8^	766.7 ± 25.2	1183.3 ± 129.0	1620.0 ± 95.4	786.7 ± 65.1	1230.0 ± 434.9	1886.7 ± 238.0
	1.0 × 10^9^	713.3 ± 15.3	793.3 ± 45.1	870.0 ± 55.7	740.0 ± 10.0	773.3 ± 145.7	830.0 ± 70.0

^a^ The counts of *S. mutans* or *P. gingivalis* after coincubation with different lactic acid bacteria (LAB) strains were significantly different, *p* < 0.01. ^b^ The counts of *S. mutans* or *P. gingivalis* after coincubation with the same LAB strain at different concentrations were significantly different, *p* < 0.01. ^c^ The counts of *S. mutans* or *P. gingivalis* after coincubation with the same LAB strain at different times were significantly different, *p* < 0.01.

**Table 2:** Counts of *S. mutans* and *P. gingivalis* after coincubation with 5 test strains within two minutes.

Strains of Lactic Acid Bacteria ^a^	Conc. of LAB (cells/mL) ^b^	Counts of *S. mutans*	Counts of *P. gingivalis*
*Lb. paracasei* 111	1.0 × 10^7^	11.7 ± 3.5	1083.3 ± 92.9
	1.0 × 10^8^	10.7 ± 2.5	880.0 ± 65.6
	1.0 × 10^9^	5.7 ± 2.5	756.7 ± 35.1
*Lb. paracasei* 127	1.0 × 10^7^	83.7 ± 12.5	80.7 ± 11.2
	1.0 × 10^8^	69.0 ± 9.2	68.0 ± 3.0
	1.0 × 10^9^	60.3 ± 9.4	61.0 ± 5.0
*Lb. paracasei* 141	1.0 × 10^7^	1023.3 ± 70.9	13.7 ± 9.8
	1.0 × 10^8^	886.7 ± 20.8	15.0 ± 3.0
	1.0 × 10^9^	710.0 ± 81.8	9.3 ± 2.5
*Llb. salivarius* 285	1.0 × 10^7^	896.7 ± 40.4	22.7 ± 2.5
	1.0 × 10^8^	826.7 ± 61.1	21.3 ± 6.5
	1.0 × 10^9^	760.0 ± 26.4	15.0 ± 1.0
*Llb. salivarius* 296	1.0 × 10^7^	840.0 ± 55.7	102.3 ± 5.1
	1.0 × 10^8^	766.7 ± 20.8	42.0 ± 7.0
	1.0 × 10^9^	720.0 ± 17.3	18.0 ± 3.6

^a^ The counts of *S. mutans* or *P. gingivalis* after coincubation with different LAB strains were significantly different, *p* < 0.01. ^b^ The counts of *S. mutans* or *P. gingivalis* after coincubation with the same LAB strain at different concentrations were significantly different, *p* < 0.01.

**Table 3:** The coaggregation values (percent) of heat-inactivated test LAB with two oral pathogen strains.

Strains of Lactic Acid Bacteria ^a^	*S. mutans*	*P. gingivalis*	*Lb. Paracasei* 141
*Lb. paracasei* 111	62.25 ± 3.46	11.47 ± 0.15	7.63 ± 0.43 *
*Lb. paracasei* 127	34.50 ± 2.65	30.133 ± 2.16	-
*Lb. paracasei* 141	17.33 ± 4.80	58.97 ± 1.70	-
*Llb. salivarius* 285	15.93 ± 2.86	57.33 ± 0.80	-
*Llb. salivarius* 296	18.10 ± 0.70	59.27 ± 0.91	-

^a^ The coagglutination value of heated test strains and oral pathogen (*S. mutans* or *P. gingivalis*) were significantly different among the test strains, *p* < 0.01. * According to the data, *Lb. paracasei* 111 and Lb. Paracasei 141 revealed a very insignificant coaggregation effect.

**Table 4:** The optimal culture conditions for *Lb. paracasei* 111 and *Lb. paracasei* 141.

Strain	Medium (*w/w*)			Culture Temperature	Controlled pH	Amount of Starter(*v/v*)	Speed of Feed ((*v/v*)/h)
	Skim Milk Powder	Glucose	Growth Factor				
*Lb. paracasei* 111	10%	2.0%	2.0%	37 ℃	5.8	7.0%	10%
*Lb. paracasei* 141	8%	3.0%	2.5%	35 ℃	6.3	5.0%	9%

**Table 5:** Freeze-drying powders of *Lb. paracasei* 111 and *Lb. paracasei* 141 prepared by culturing under optimal conditions.

Strain	Powder Weight (Gram)	Loss on Drying (%, *w/w*)	Bacterial Biomass (Cells/g)
*Lb. paracasei* 111	403	2.9 ± 0.3	1.47 × 10^11^ ± 0.31
*Lb. paracasei* 141	421	3.1 ± 0.2	1.12 × 10^11^ ± 0.26

**Table 6:** The composition of tablets was developed based on tests.

Ingredients ^a^	*Lb. paracasei* 111	*Lb. paracasei* 141	Maltodextrin	Sorbitol	Xylitol	Silicon Dioxide	Flavor
Ratio ^b^	10%	10%	36%	15%	15%	13%	1%

^a^ All ingredients were powder form. ^b^ Ratio was *w/w*.

**Table 7:** The examined data of tablets.

Item	Inhibitory Effect on *S. mutans*	Inhibitory Effect on *P. gingivalis*	Disintegration Time	Flavor Quality ^b^	Hardness	Loss on Drying
Data	5 ± 3 ^a^	11 ± 2 ^a^	500 ± 7 sec	7.8 ± 0.2	22.1 ± 0.5 kg	4.32 ± 0.11%

^a^ Counts of *S. mutans* or *P. gingivalis* after coincubation with tablet powder solution. ^b^ 9-point hedonic score.

**Table 8:** The inhibition of periodontal pathogens in volunteers’ oral cavity.

Periodontal Diseases	Time	Placebo, n = 20	Product, n = 20	*p* Value ^a^
Positive	Baseline	10	8	0.404
Negative		10	12	
Positive	4 weeks	9	1	0.003 **
Negative		11	19	
Positive	8 weeks	9	1	0.003 **
Negative		11	19	
*p* value ^b^		0.999	0.016 *	
*p* value ^c^		1.000	0.016 *	

^a^ The *p* value is the difference between placebo and product groups, analyzed by Fisher’s exact test. ^b^ The *p* value is the difference between baseline and the data of 4 weeks, analyzed by McNemar’s test. ^c^ The *p* value is the difference between baseline and the data of 8 weeks, analyzed by McNemar’s test. * *p* < 0.05, ** *p* < 0.01.

**Table 9:** The improvement of probing pocket depth (PPD) of volunteers.

Index	Score (mm)	Treating Time	Placebo, n = 20	Product, n = 20	*p* Value ^a^
PPD	<5.0	Baseline	0	1	0.311
	≥5.0		20	19	
	<5.0	4 weeks	5	6	0.723
	≥5.0		15	14	
	<5.0	8 weeks	10	16	0.047 *
	≥5.0		10	4	

^a^*p* value is the difference between placebo and product groups, analyzed by Fisher’s exact test. * *p* < 0.05.

**Table 10:** The improvement from baseline of halitosis of volunteers.

Halitosis	Time	Placebo, n = 20	Product, n = 20	*p* Value ^a^
Yes	4 weeks	3	9	0.038 *
No		17	11	
Yes	8 weeks	6	11	0.110
No		14	9	

^a^ The *p* value is the difference between placebo and product groups, analyzed by Fisher’s exact test. Improvement of halitosis was defined as the ratio between baseline and the data of 4th and 8th weeks ≥ 1.1. * *p* < 0.05. The improvement from baseline of halitosis of volunteers.

## Data Availability

Data is contained within this article and the supplementary material.

## Supplementary Materials

The following are available online at https://www.mdpi.com/2075-1729/11/4/268/s1, Figure S1: The co-aggregation reaction of heat-inactivated test strains and oral pathogens, Table S1: The results of Anti-oral pathogenic bacteria testing on bacteriocin crude extracts of 450 LAB strains (unit: mm), Table S2: The factors and levels per factor of the first experiments for Lb. paracasei 111 and Lb. paracasei 141, Table S3: The design of experiments and the results of the first experiment of optimal culture media and conditions for Lb. paracasei 111 (unit: 1.0 × 10^8^ cfu/mL), Table S4: The factors and levels per factor of the second experiments for Lb. paracasei 111 and Lb. paracasei 141, Table S5: The design of experiment and the results of the second experiment of optimal culture media and culture conditions for Lb. paracasei 111 (unit: 1.0 × 10^8^ cfu/mL), Table S6. The design of experiment and the results of the first experiment of optimal culture media and culture conditions for Lb. paracasei 141 (unit: 1.0 × 10^8^ cfu/mL), Table S7: The design of experiment and the results of the second experiment of optimal culture media and culture conditions for Lb. paracasei 141 (unit: 1.0 × 10^8^ cfu/mL), Table S8: The predicted product self-life at 25 °C and the actual measured quality value at this shelf-life.
