# Supplementary material for: Developing Lactic Acid Bacteria as an Oral Healthy Food
Source: Life (Basel). 2021 Mar 24;11(4):268. doi: 10.3390/life11040268 (PMC8064088; doi:10.3390/life11040268)
Supplement: Supplementary file 1 [file life-11-00268-s001.pdf]

Supplementary materials

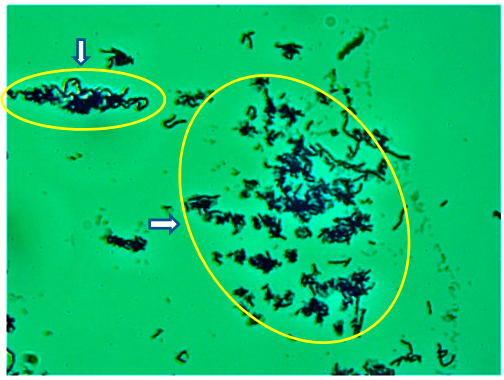

A

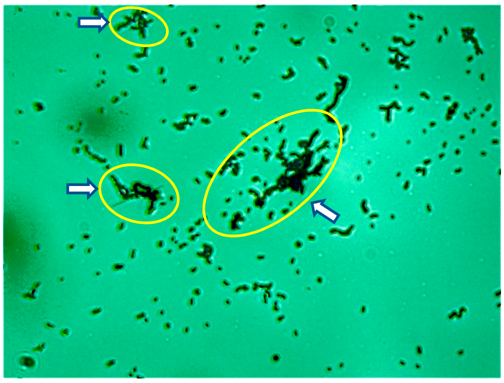

B

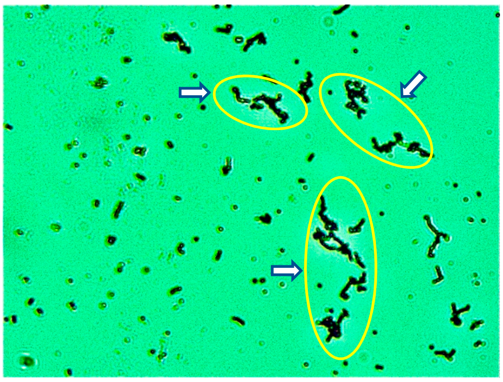

C

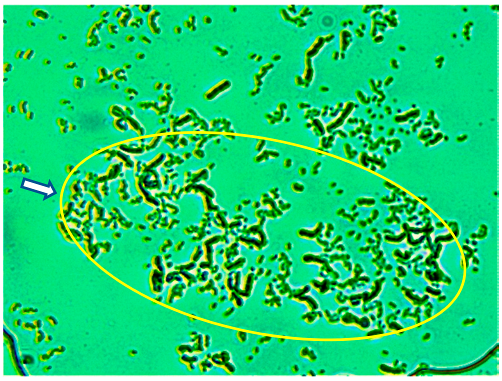

D

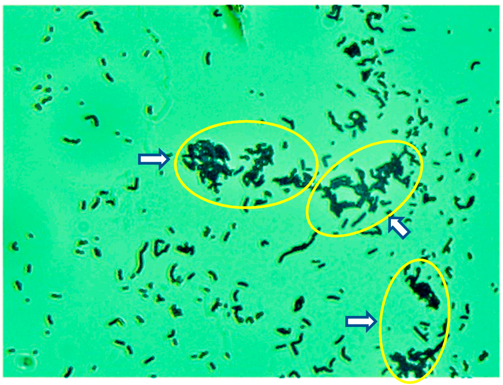

E

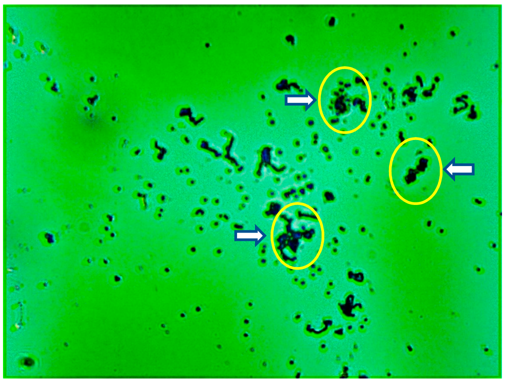

F

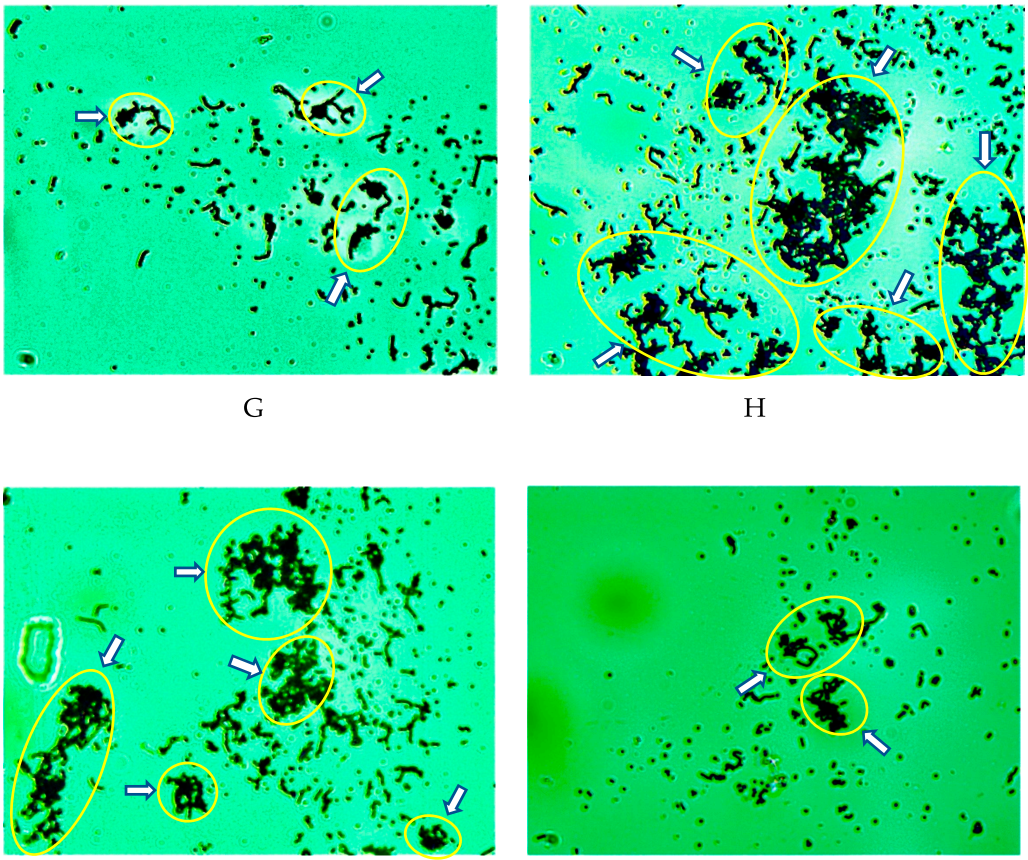

G H

I J

Fig S1. The co-aggregation reaction of heat-inactivated test strains and oral pathogens.

A was *Lacticaseibacillus (Lb). paracasei* 111 & *Streptococcus mutans*. B was *Lb. paracasei* 127 & *S. mutans*. C was *Lb. paracasei* 141 & *S. mutans*. D was *Lb. salivarius* 285 & *S. mutans*. E was *Lb. salivarius* 296 & *S. mutans*. F was *Lb. paracasei* 111 & *Porphyromonas gingivalis*. G was *Lb. paracasei* 127 & *P. gingivalis*. H was *Lb. paracasei* 141 & *P. gingivalis*. I was *Lb. salivarius* 285 & *P. gingivalis*. J was *Lb. salivarius* 296 & *Pb. gingivalis*.

Arrows and circles indicate co-coagulated objects.

Table S1. The results of Anti-oral pathogenic bacteria testing on bacteriocin crude extracts of 450 LAB strains (unit: mm).

**Code in laboratory**	**LAB strains**		**Origin**	**Code in this study**	**Zone of inhibition for** *S. mutans* [a]			**Zone of inhibition for** *P. gingivalis* [b]		
	Previous name	Latest name								
LUC-La001	*Lactobacillus acidophilus*	*Lactobacillus acidophilus*	Baby feces	1	ND	ND	ND	8.58	8.47	8.11
LUC-La002	*L. acidophilus*	*L. acidophilus*	Baby feces	2	ND	ND	ND	9.02	9.04	9.55
LUC-La003	*L. acidophilus*	*L. acidophilus*	Baby feces	3	ND	ND	ND	9.53	10.02	10.91
LUC-La004	*L. acidophilus*	*L. acidophilus*	Baby feces	4	ND	ND	ND	8.74	8.87	9.01
LUC-A0005	*L. acidophilus*	*L. acidophilus*	Baby feces	5	ND	ND	ND	10.02	10.62	10.01
LUC-La006	*L. acidophilus*	*L. acidophilus*	Human intestine	6	ND	ND	ND	9.25	9.45	9.89
LUC-La007	*L. acidophilus*	*L. acidophilus*	Human intestine	7	ND	ND	ND	10.2	10.06	10.65
LUC-La008	*L. acidophilus*	*L. acidophilus*	Human intestine	8	ND	ND	ND	8.55	8.94	9.04

LUC-La009	*L. acidophilus*	*L. acidophilus*	Human intestine	9	ND	ND	ND	11.01	10.89	10.21
LUC-La010	*L. acidophilus*	*L. acidophilus*	Baby feces	10	ND	ND	ND	8.84	8.94	8.35
LUC-La011	*L. acidophilus*	*L. acidophilus*	Human intestine	11	ND	ND	ND	10.41	10.82	10.39
LUC-La012	*L. acidophilus*	*L. acidophilus*	Human intestine	12	ND	ND	ND	9.36	10.23	9.20
LUC-La013	*L. acidophilus*	*L. acidophilus*	Human intestine	13	ND	ND	ND	10.28	10.32	10.69
LUC-La014	*L. acidophilus*	*L. acidophilus*	Human vagina	14	ND	ND	ND	14.62	14.35	14.00
LUC-La015	*L. acidophilus*	*L. acidophilus*	Human vagina	15	ND	ND	ND	14.31	14.85	14.73
LUC-La016	*L. acidophilus*	*L. acidophilus*	Baby feces	16	ND	ND	ND	14.21	14.38	14.52
LUC-La017	*L. acidophilus*	*L. acidophilus*	Human vagina	17	ND	ND	ND	12.11	12.41	12.97
LUC-La018	*L. acidophilus*	*L. acidophilus*	Human vagina	18	ND	ND	ND	12.00	12.18	12.35
LUC-A0019	*L. acidophilus*	*L. acidophilus*	Healthy food	19	ND	ND	ND	12.32	12.57	12.62
LUC-La020	*L. acidophilus*	*L. acidophilus*	Baby feces	20	ND	ND	ND	12.05	12.95	12.37
LUC-La021	*L. acidophilus*	*L. acidophilus*	Human intestine	21	ND	ND	ND	11.21	11.52	11.99
LUC-La022	*L. acidophilus*	*L. acidophilus*	Human intestine	22	ND	ND	ND	11.00	11.30	11.86
LUC-La023	*L. acidophilus*	*L. acidophilus*	Healthy food	23	ND	ND	ND	ND	ND	ND
LUC-La024	*L. acidophilus*	*L. acidophilus*	Baby feces	24	ND	ND	ND	ND	ND	ND
LUC-La025	*L. acidophilus*	*L. acidophilus*	Baby feces	25	ND	ND	ND	12.85	12.55	12.61
LUC-La026	*L. acidophilus*	*L. acidophilus*	Human vagina	26	ND	ND	ND	12.02	11.32	11.14
LUC-La027	*L. acidophilus*	*L. acidophilus*	Healthy food	27	ND	ND	ND	14.62	14.35	14
LUC-La028	*L. acidophilus*	*L. acidophilus*	Healthy food	28	ND	ND	ND	13.89	13.79	13.73
LUC-Lb001	*Lactobacillus delbrueckii* subsp. *bulgaricus*	*Lactobacillus delbrueckii* subsp. *bulgaricus*	Yogurt	29	ND	ND	ND	14.77	14.27	14.62
LUC-Lb002	*L. delbrueckii* subsp. *bulgaricus*	*L. delbrueckii* subsp. *bulgaricus*	Yogurt	30	ND	ND	ND	ND	ND	ND
LUC-Lb003	*L. delbrueckii* subsp. *bulgaricus*	*L. delbrueckii* subsp. *bulgaricus*	Yogurt	31	ND	ND	ND	10.63	13.74	9.55
LUC-Lb004	*L. delbrueckii* subsp. *bulgaricus*	*L. delbrueckii* subsp. *bulgaricus*	Yogurt	32	ND	ND	ND	10.34	10.36	10.96
LUC-Lb005	*L. delbrueckii* subsp. *bulgaricus*	*L. delbrueckii* subsp. *bulgaricus*	Fermented milk	33	ND	ND	ND	9.88	9.32	10.08
LUC-Lb006	*L. delbrueckii* subsp. *bulgaricus*	*L. delbrueckii* subsp. *bulgaricus*	Fermented milk	34	ND	ND	ND	9.93	9.63	9.23
LUC-Lb007	*L. delbrueckii* subsp. *bulgaricus*	*L. delbrueckii* subsp. *bulgaricus*	Fermented milk	35	ND	ND	ND	12.01	12.47	12.55

LUC-Lb008	*L. delbrueckii* subsp. *bulgaricus*	*L. delbrueckii* subsp. *bulgaricus*	Yogurt	36	ND	ND	ND	13.04	13.21	13.05
LUC-Lb009	*L. delbrueckii* subsp. *bulgaricus*	*L. delbrueckii* subsp. *bulgaricus*	Yogurt	37	ND	ND	ND	11.92	11.89	11.82
LUC-Lb010	*L. delbrueckii* subsp. *bulgaricus*	*L. delbrueckii* subsp. *bulgaricus*	Yogurt	38	ND	ND	ND	25.33	20.82	20.94
LUC-Lb011	*L. delbrueckii* subsp. *bulgaricus*	*L. delbrueckii* subsp. *bulgaricus*	Yogurt	39	ND	ND	ND	ND	ND	ND
LUC-Lb012	*L. delbrueckii* subsp. *bulgaricus*	*L. delbrueckii* subsp. *bulgaricus*	Yogurt	40	ND	ND	ND	ND	ND	ND
LUC-Lb013	*L. delbrueckii* subsp. *bulgaricus*	*L. delbrueckii* subsp. *bulgaricus*	Yogurt	41	ND	ND	ND	ND	ND	ND
LUC-Lb014	*L. delbrueckii* subsp. *bulgaricus*	*L. delbrueckii* subsp. *bulgaricus*	Yogurt	42	ND	ND	ND	28.87	28.92	28.30
LUC-Lb015	*L. delbrueckii* subsp. *bulgaricus*	*L. delbrueckii* subsp. *bulgaricus*	Yogurt	43	ND	ND	ND	30.10	30.99	30.68
LUC-Lb016	*L. delbrueckii* subsp. *bulgaricus*	*L. delbrueckii* subsp. *bulgaricus*	Yogurt	44	ND	ND	ND	29.32	29.87	29.90
LUC-Lb017	*L. delbrueckii* subsp. *bulgaricus*	*L. delbrueckii* subsp. *bulgaricus*	Yogurt	45	ND	ND	ND	32.01	31.62	31.56
LUC-Lbr001	*Lactobacillus casei*	*Lacticaseibacillus* (*Lb*) *casei*	Fermented milk	46	ND	ND	ND	18.56	18.02	18.83
LUC-Lbr002	*L. casei*	*Lb. casei*	Fermented milk	47	ND	ND	ND	14.01	14.35	14.80
LUC-Lbr003	*L. casei*	*Lb. casei*	Fermented milk	48	ND	ND	ND	12.87	12.98	12.39
LUC-Lbr004	*L. casei*	*Lb. casei*	Fermented milk	49	ND	ND	ND	11.85	11.32	11.57
LUC-Lbr005	*L. casei*	*Lb. casei*	Fermented milk	50	ND	ND	ND	14.18	14.05	13.96
LUC-Lbr006	*L. casei*	*Lb. casei*	Cheese	51	ND	ND	ND	ND	ND	ND
LUC-Lbr007	*L. casei*	*Lb. casei*	Cheese	52	ND	ND	ND	13.88	13.61	13.94
LUC-Lbr008	*L. casei*	*Lb. casei*	Cheese	53	ND	ND	ND	ND	ND	ND
LUC-Lbr009	*L. casei*	*Lb. casei*	Cheese	54	ND	ND	ND	12.01	12.55	13.02
LUC-Lbr010	*L. casei*	*Lb. casei*	Cheese	55	ND	ND	ND	ND	ND	ND
LUC-Lbr011	*L. casei*	*Lb. casei*	Cheese	56	ND	ND	ND	11.03	11.10	10.84
LUC-Lbr012	*L. casei*	*Lb. casei*	Yogurt	57	ND	ND	ND	10.28	10.09	9.98
LUC-Lbr013	*L. casei*	*Lb. casei*	Yogurt	58	ND	ND	ND	13.01	13.54	13.62
LUC-Lbr014	*L. casei*	*Lb. casei*	Yogurt	59	ND	ND	ND	ND	ND	ND
LUC-Lbr015	*L. casei*	*Lb. casei*	Yogurt	60	13.96	14.49	13.8	14.77	14.20	14.62
LUC-Lbr016	*L. casei*	*Lb. casei*	Healthy food	61	ND	ND	ND	ND	ND	ND

LUC-Lc001	*L. casei*	*Lb. casei*	Healthy food	62	ND	ND	ND	10.20	10.65	10.44
LUC-Lc002	*L. casei*	*Lb. casei*	Healthy food	63	ND	ND	ND	10.98	11.36	10.87
LUC-Lc003	*L. casei*	*Lb. casei*	Healthy food	64	ND	ND	ND	9.81	9.78	9.24
LUC-Lc004	*L. casei*	*Lb. casei*	Raw milk	65	ND	ND	ND	12.99	12.60	12.71
LUC-Lc005	*L. casei*	*Lb. casei*	Raw milk	66	ND	ND	ND	ND	ND	ND
LUC-Lc006	*L. casei*	*Lb. casei*	Raw milk	67	ND	ND	ND	13.21	13.84	13.86
LUC-Lc007	*L. casei*	*Lb. casei*	Raw milk	68	ND	ND	ND	13.52	13.24	13.77
LUC-Lc008	*L. casei*	*Lb. casei*	Healthy food	69	12.56	13.45	11.96	12.79	11.58	11.32
LUC-Lc009	*L. casei*	*Lb. casei*	Healthy food	70	ND	ND	ND	ND	ND	ND
LUC-Lc010	*L. casei*	*Lb. casei*	Baby feces	71	ND	ND	ND	ND	ND	ND
LUC-Lc011	*L. casei*	*Lb. casei*	Baby feces	72	ND	ND	ND	ND	ND	ND
LUC-Lc012	*L. casei*	*Lb. casei*	Baby feces	73	ND	ND	ND	9.56	9.88	9.62
LUC-Lc013	*L. casei*	*Lb. casei*	Healthy food	74	13.04	12.16	12.05	12.73	14.82	12.15
LUC-Lc014	*L. casei*	*Lb. casei*	Healthy food	75	ND	ND	ND	ND	ND	ND
LUC-Lc015	*L. casei*	*Lb. casei*	Healthy food	76	ND	ND	ND	ND	ND	ND
LUC-Lc016	*L. casei*	*Lb. casei*	Yogurt	77	ND	ND	ND	ND	ND	ND
LUC-Lc017	*L. casei*	*Lb. casei*	Yogurt	78	ND	ND	ND	ND	ND	ND
LUC-Lc018	*L. casei*	*Lb. casei*	Yogurt	79	ND	ND	ND	ND	ND	ND
LUC-Lc019	*L. casei*	*Lb. casei*	Sour cream	80	ND	ND	ND	11.02	11.54	11.31
LUC-Lc020	*L. casei*	*Lb. casei*	Sour cream	81	ND	ND	ND	10.91	10.32	10.83
LUC-Lc021	*L. casei*	*Lb. casei*	Sour cream	82	ND	ND	ND	12.14	12.73	12.52
LUC-Lc022	*L. casei*	*Lb. casei*	Sour cream	83	ND	ND	ND	ND	ND	ND
LUC-Lc023	*L. casei*	*Lb. casei*	Sour cream	84	ND	ND	ND	ND	ND	ND
LUC-Lc024	*L. casei*	*Lb. casei*	Baby feces	85	ND	ND	ND	ND	ND	ND
LUC-Lc025	*L. casei*	*Lb. casei*	Baby feces	86	ND	ND	ND	10.69	10.31	10.35
LUC-Lc026	*L. casei*	*Lb. casei*	Baby feces	87	ND	ND	ND	10.99	10.68	10.12
LUC-Lc027	*L. casei*	*Lb. casei*	Baby feces	88	ND	ND	ND	11.93	11.99	12.08
LUC-Lc028	*Lactobacillus brevis*	*Levilactobacillus brevis*	Sauerkraut	89	9.03	11.63	8.95	ND	ND	ND
LUC-Lc029	*L. brevis*	*Lb. brevis*	Sauerkraut	90	ND	ND	ND	ND	ND	ND
LUC-Lc030	*L. brevis*	*Lb. brevis*	Sauerkraut	91	ND	ND	ND	12.73	14.82	12.15
LUC-Lc031	*L. brevis*	*Lb. brevis*	Sauerkraut	92	ND	ND	ND	12.87	12.98	12.39
LUC-Lc032	*L. brevis*	*Lb. brevis*	Sauerkraut	93	ND	ND	ND	ND	ND	ND
LUC-Lc033	*L. brevis*	*Lb. brevis*	Pickles	94	ND	ND	ND	ND	ND	ND
LUC-Lc034	*L. brevis*	*Lb. brevis*	Pickles	95	ND	ND	ND	ND	ND	ND

LUC-Lc035	*L. brevis*	*Lb. brevis*	Pickles	96	ND	ND	ND	ND	ND	ND
LUC-Lc036	*L. brevis*	*Lb. brevis*	Pickles	97	ND	ND	ND	ND	ND	ND
LUC-Lc037	*L. brevis*	*Lb. brevis*	Pickles	98	ND	ND	ND	13.73	14.82	13.15
LUC-Lc038	*L. brevis*	*Lb. brevis*	Pickles	99	ND	ND	ND	ND	ND	ND
LUC-Lc039	*L. brevis*	*Lb. brevis*	Healthy food	100	ND	ND	ND	11.79	12.52	11.94
LUC-Lc040	*L. brevis*	*Lb. brevis*	Healthy food	101	ND	ND	ND	ND	ND	ND
LUC-Lc041	*L. brevis*	*Lb. brevis*	Healthy food	102	ND	ND	ND	13.74	13.82	14.11
LUC-Lc042	*L. brevis*	*Lb. brevis*	Healthy food	103	ND	ND	ND	ND	ND	ND
LUC-Lc043	*L. brevis*	*Lb. brevis*	Healthy food	104	ND	ND	ND	ND	ND	ND
LUC-Lc044	*L. brevis*	*Lb. brevis*	Healthy food	105	ND	ND	ND	ND	ND	ND
LUC-Lc045	*L. brevis*	*Lb. brevis*	Healthy food	106	ND	ND	ND	12.91	13.52	13.22
LUC-Lpc001	*Lactobacillus paracasei*	*Lacticaseibacillus paracasei*	Healthy food	107	ND	ND	ND	18.21	18.34	19.05
LUC-Lpc 002	*L. paracasei*	*Lb. paracasei*	Healthy food	108	ND	ND	ND	ND	ND	ND
LUC-Lpc003	*L. paracasei*	*Lb. paracasei*	Healthy food	109	ND	ND	ND	26.32	25.99	26.10
LUC-Lpc004	*L. paracasei*	*Lb. paracasei*	Healthy food	110	ND	ND	ND	ND	ND	ND
LUC-Lpc005	*L. paracasei*	*Lb. paracasei*	Healthy food	111	14.05	14.28	13.95	ND	ND	ND
LUC-Lpc006	*L. paracasei*	*Lb. paracasei*	Healthy food	112	ND	ND	ND	ND	ND	ND
LUC-Lpc007	*L. paracasei*	*Lb. paracasei*	Healthy food	113	ND	ND	ND	21.17	21.58	21.07
LUC-Lpc008	*L. paracasei*	*Lb. paracasei*	Healthy food	114	ND	ND	ND	22.42	22.35	22.82
LUC-Lpc009	*L. paracasei*	*Lb. paracasei*	Healthy food	115	ND	ND	ND	27.78	27.31	27.62
LUC-Lpc010	*L. paracasei*	*Lb. paracasei*	Healthy food	116	ND	ND	ND	27.05	27.12	27.56
LUC-Lpc011	*L. paracasei*	*Lb. paracasei*	Healthy food	117	ND	ND	ND	27.73	27.01	27.32
LUC-Lpc012	*L. paracasei*	*Lb. paracasei*	Healthy food	118	ND	ND	ND	24.11	24.32	24.55
LUC-Lpc013	*L. paracasei*	*Lb. paracasei*	Healthy food	119	ND	ND	ND	21.07	21.68	21.05
LUC-Lpc014	*L. paracasei*	*Lb. paracasei*	Healthy food	120	ND	ND	ND	25.11	25.66	25.41
LUC-Lpc015	*L. paracasei*	*Lb. paracasei*	Healthy food	121	ND	ND	ND	17.05	17.23	17.65
LUC-Lpc016	*L. paracasei*	*Lb. paracasei*	Healthy food	122	ND	ND	ND	31.56	30.96	30.13
LUC-Lpc019	*L. paracasei*	*Lb. paracasei*	Baby feces	123	ND	ND	ND	27.19	27.86	27.37
LUC-Lpc020	*L. paracasei*	*Lb. paracasei*	Baby feces	124	ND	ND	ND	10.21	10.29	10.85
LUC-Lpc021	*L. paracasei*	*Lb. paracasei*	Baby feces	125	ND	ND	ND	11.88	11.32	11.95
LUC-Lpc022	*L. paracasei*	*Lb. paracasei*	Baby feces	126	ND	ND	ND	30.22	30.85	30.07
LUC-Lpc023	*L. paracasei*	*Lb. paracasei*	Baby feces	127	13.36	12.66	12.55	23.58	25.60	23.47
LUC-Lpc024	*L. paracasei*	*Lb. paracasei*	Human milk	128	ND	ND	ND	18.04	18.92	18.32
LUC-Lpc025	*L. paracasei*	*Lb. paracasei*	Human milk	129	ND	ND	ND	16.66	16.89	16.05

LUC-Lpc026	*L. paracasei*	*Lb. paracasei*	Human milk	130	ND	ND	ND	16.53	16.74	16.62
LUC-Lpc027	*L. paracasei*	*Lb. paracasei*	Human milk	131	ND	ND	ND	22.11	22.62	22.51
LUC-Lpc028	*L. paracasei*	*Lb. paracasei*	Human milk	132	ND	ND	ND	15.21	15.81	15.36
LUC-Lpc029	*L. paracasei*	*Lb. paracasei*	Human intestine	133	ND	ND	ND	16.03	16.66	16.29
LUC-Lpc030	*L. paracasei*	*Lb. paracasei*	Human intestine	134	ND	ND	ND	17.87	17.98	17.04
LUC-Lpc031	*L.paracasei*	*Lb. paracasei*	Human intestine	135	ND	ND	ND	24.24	24.35	24.15
LUC-Lpc032	*L. paracasei*	*Lb. paracasei*	Human intestine	136	ND	ND	ND	14.29	14.40	14.17
LUC-Lpc033	*L. paracasei*	*Lb. paracasei*	Human intestine	137	ND	ND	ND	17.02	17.39	18.24
LUC-Lpc034	*L. paracasei*	*Lb. paracasei*	Healthy food	138	ND	ND	ND	26.45	25.12	24.76
LUC-Lpc035	*L. paracasei*	*Lb. paracasei*	Healthy food	139	ND	ND	ND	28.31	28.43	29.55
LUC-Lpc036	*L. paracasei*	*Lb. paracasei*	Healthy food	140	ND	ND	ND	29.38	29.64	28.08
LUC-Lpc037	*L. paracasei*	*Lb. paracasei*	Healthy food	141	ND	ND	ND	32.11	33.53	32.05
LUC-Lpc038	*L. paracasei*	*Lb. paracasei*	Healthy food	142	ND	ND	ND	19.95	19.22	19.65
LUC-Lpc039	*L. paracasei*	*Lb. paracasei*	Healthy food	143	ND	ND	ND	25.61	25.38	25.99
LUC-Lpc040	*L. paracasei*	*Lb. paracasei*	Human oral	144	ND	ND	ND	23.19	23.88	23.69
LUC-Lpc041	*L. paracasei*	*Lb. paracasei*	Human oral	145	ND	ND	ND	27.88	27.59	26.41
LUC-Lpc042	*L. paracasei*	*Lb. paracasei*	Human oral	146	ND	ND	ND	22.78	23.60	23.32
LUC-Lpc043	*L. paracasei*	*Lb. paracasei*	Human oral	147	14.62	13.97	13.84	ND	ND	ND
LUC-Lpc044	*L. paracasei*	*Lb. paracasei*	Human oral	148	ND	ND	ND	10.33	10.41	10.57
LUC-Lpc045	*L. paracasei*	*Lb. paracasei*	Silage	149	ND	ND	ND	15.61	15.94	15.22
LUC-Lpc046	*L. paracasei*	*Lb. paracasei*	Silage	150	ND	ND	ND	9.67	9.69	9.54
LUC-Lf001	*Lactobacillus fermentum*	*Limosilactobacillus fermentum*	Fermenting meat	151	ND	ND	ND	9.32	9.21	9.01
LUC-Lf002	*L. fermentum*	*L. fermentum*	Fermenting meat	152	ND	ND	ND	8.84	8.56	8.33
LUC-Lf003	*L. fermentum*	*L. fermentum*	Fermenting meat	153	ND	ND	ND	8.15	8.14	8.24
LUC-Lf004	*L. fermentum*	*L. fermentum*	Fermenting meat	154	ND	ND	ND	ND	ND	ND
LUC-Lf005	*L. fermentum*	*L. fermentum*	Fermenting meat	155	ND	ND	ND	ND	ND	ND
LUC-Lf006	*L. fermentum*	*L. fermentum*	Fermenting meat	156	ND	ND	ND	ND	ND	ND
LUC-Lf007	*L. fermentum*	*L. fermentum*	Sausage	157	ND	ND	ND	ND	ND	ND
LUC-Lf008	*L. fermentum*	*L. fermentum*	Sausage	158	ND	ND	ND	ND	ND	ND
LUC-Lf009	*L. fermentum*	*L. fermentum*	Sausage	159	ND	ND	ND	ND	ND	ND
LUC-Lf010	*L. fermentum*	*L. fermentum*	Sausage	160	ND	ND	ND	7.98	7.65	7.81
LUC-Lf011	*L. fermentum*	*L. fermentum*	Sausage	161	ND	ND	ND	8.03	8.23	8.11
LUC-Lf012	*L. fermentum*	*L. fermentum*	Pickle	162	ND	ND	ND	9.65	9.32	9.13
LUC-Lf013	*L. fermentum*	*L. fermentum*	Pickle	163	ND	ND	ND	ND	ND	ND

LUC-Lf014	*L. fermentum*	*L. fermentum*	Pickle	164	ND	ND	ND	ND	ND	ND
LUC-Lf015	*L. fermentum*	*L. fermentum*	Pickle	165	ND	ND	ND	ND	ND	ND
LUC-Lf016	*L. fermentum*	*L. fermentum*	Pickle	166	ND	ND	ND	12.31	12.87	13.05
LUC-Lf017	*L. fermentum*	*L. fermentum*	Pickle	167	ND	ND	ND	ND	ND	ND
LUC-Lf018	*L. fermentum*	*L. fermentum*	Sauerkraut	168	ND	ND	ND	ND	ND	ND
LUC-Lf019	*L. fermentum*	*L. fermentum*	Sauerkraut	169	ND	ND	ND	14.05	14.16	13.98
LUC-Lf020	*L. fermentum*	*L. fermentum*	Sauerkraut	170	ND	ND	ND	ND	ND	ND
LUC-Lf021	*L. fermentum*	*L. fermentum*	Sauerkraut	171	ND	ND	ND	ND	ND	ND
LUC-Ll001	*Lactobacillus delbrueckii* subsp. *lactis*	*Lactobacillus delbrueckii* subsp. *lactis*	Raw milk	172	ND	ND	ND	16.32	16.33	16.84
LUC-Ll002	*L. delbrueckii* subsp. *lactis*	*L. delbrueckii* subsp. *lactis*	Raw milk	173	ND	ND	ND	11.47	12.53	11.05
LUC-Ll003	*L. delbrueckii* subsp. *lactis*	*L. delbrueckii* subsp. *lactis*	Raw milk	174	ND	ND	ND	15.02	16.01	15.57
LUC-Ll0004	*L. delbrueckii* subsp. *lactis*	*L. delbrueckii* subsp. *lactis*	Raw milk	175	ND	ND	ND	10.96	10.69	10.33
LUC-Ll005	*L. delbrueckii* subsp. *lactis*	*L. delbrueckii* subsp. *lactis*	Raw milk	176	ND	ND	ND	9.94	10.12	9.83
LUC-Ll006	*L. delbrueckii* subsp. *lactis*	*L. delbrueckii* subsp. *lactis*	Cheese	177	ND	ND	ND	8.74	8.55	8.92
LUC-Ll007	*L. delbrueckii* subsp. *lactis*	*L. delbrueckii* subsp. *lactis*	Cheese	178	ND	ND	ND	8.21	8.02	8.69
LUC-Ll008	*L. delbrueckii* subsp. *lactis*	*L. delbrueckii* subsp. *lactis*	Cheese	179	ND	ND	ND	7.91	7.95	8.09
LUC-Ll009	*L. delbrueckii* subsp. *lactis*	*L. delbrueckii* subsp. *lactis*	Cheese	180	ND	ND	ND	ND	ND	ND
LUC-Lg001	*Lactobacillus gasseri*	*Lactobacillus gasseri*	Human vagina	181	ND	ND	ND	ND	ND	ND
LUC-Lg002	*L. gasseri*	*L. gasseri*	Human vagina	182	ND	ND	ND	24.07	25.60	23.86
LUC-Lg003	*L. gasseri*	*L. gasseri*	Human vagina	183	ND	ND	ND	ND	ND	ND
LUC-Lg004	*L. gasseri*	*L. gasseri*	Human vagina	184	ND	ND	ND	10.23	11.00	11.08
LUC-Lg005	*L. gasseri*	*L. gasseri*	Human vagina	185	ND	ND	ND	11.25	10.18	10.65
LUC-Lg006	*L. gasseri*	*L. gasseri*	Human vagina	186	ND	ND	ND	13.05	12.54	12.37
LUC-Lg007	*L. gasseri*	*L. gasseri*	Human vagina	187	ND	ND	ND	ND	ND	ND
LUC-Lg008	*L. gasseri*	*L. gasseri*	Healthy food	188	ND	ND	ND	ND	ND	ND
LUC-Lg009	*L. gasseri*	*L. gasseri*	Healthy food	189	ND	ND	ND	ND	ND	ND

LUC-Lg010	*L. gasseri*	*L. gasseri*	Healthy food	190	ND	ND	ND	9.94	10.12	9.83
LUC-Lg011	*L. gasseri*	*L. gasseri*	Healthy food	191	ND	ND	ND	11.28	11.98	11
LUC-Lg012	*L. gasseri*	*L. gasseri*	Healthy food	192	ND	ND	ND	12.01	11.22	11.98
LUC-Lh001	*Lactobacillus helveticus*	*Lactobacillus helveticus*	Cheese	193	ND	ND	ND	10.12	10.98	11.35
LUC-Lh002	*L. helveticus*	*L. helveticus*	Cheese	194	ND	ND	ND	ND	ND	ND
LUC-Lh003	*L. helveticus*	*L. helveticus*	Cheese	195	ND	ND	ND	ND	ND	ND
LUC-Lh004	*L. helveticus*	*L. helveticus*	Cheese	196	ND	ND	ND	ND	ND	ND
LUC-Lh005	*L. helveticus*	*L. helveticus*	Cheese	197	ND	ND	ND	8.14	8.42	8.67
LUC-Lh006	*L. helveticus*	*L. helveticus*	Cheese	198	ND	ND	ND	ND	ND	ND
LUC-Lh007	*L. helveticus*	*L. helveticus*	Olives	199	ND	ND	ND	7.77	7.95	7.99
LUC-Lh008	*L. helveticus*	*L. helveticus*	Olives	200	ND	ND	ND	7.64	7.66	7.92
LUC-Lh009	*L. helveticus*	*L. helveticus*	Pickles	201	ND	ND	ND	8.09	8.31	8.11
LUC-Lh010	*L. helveticus*	*L. helveticus*	Pickles	202	ND	ND	ND	9.21	9.11	8.99
LUC-Lh011	*L. helveticus*	*L. helveticus*	Cheese	203	ND	ND	ND	9.56	9.21	9.30
LUC-Lh012	*L. helveticus*	*L. helveticus*	Cheese	204	ND	ND	ND	8.05	8.22	8.32
LUC-Lh013	*L. helveticus*	*L. helveticus*	Pickles	205	ND	ND	ND	10.21	10.36	10.61
LUC-Lh014	*L. helveticus*	*L. helveticus*	Pickles	206	ND	ND	ND	7.55	7.62	7.96
LUC-Lh015	*L. helveticus*	*L. helveticus*	Fermented milk	207	ND	ND	ND	8.09	8.02	8.32
LUC-Lh016	*L. helveticus*	*L. helveticus*	Fermented milk	208	ND	ND	ND	10.41	10.06	10.31
LUC-Lh017	*L. helveticus*	*L. helveticus*	Fermented milk	209	ND	ND	ND	ND	ND	ND
LUC-Lh018	*L. helveticus*	*L. helveticus*	Fermented milk	210	ND	ND	ND	ND	ND	ND
LUC-Lh019	*L. helveticus*	*L. helveticus*	Fermented milk	211	ND	ND	ND	10.35	10.44	10.26
LUC-Lh020	*L. helveticus*	*L. helveticus*	Cheese	212	ND	ND	ND	ND	ND	ND
LUC-Lh021	*L. helveticus*	*L. helveticus*	Cheese	213	ND	ND	ND	ND	ND	ND
LUC-Lh022	*L. helveticus*	*L. helveticus*	Cheese	214	ND	ND	ND	9.81	9.23	9.07
LUC-Lh023	*L. helveticus*	*L. helveticus*	Cheese	215	ND	ND	ND	ND	ND	ND
LUC-Lh024	*L. helveticus*	*L. helveticus*	Cheese	216	11.56	11.41	11.31	10.35	10.44	10.26
LUC-Lh025	*L. helveticus*	*L. helveticus*	Raw milk	217	ND	ND	ND	ND	ND	ND
LUC-Lh026	*L. helveticus*	*L. helveticus*	Raw milk	218	ND	ND	ND	11.53	11.25	11.47
LUC-Lh027	*L. helveticus*	*L. helveticus*	Kefir	219	ND	ND	ND	10.02	10.39	10.03
LUC-Lh028	*L. helveticus*	*L. helveticus*	Kefir	220	ND	ND	ND	12.21	16.99	12.73
LUC-Lh029	*L. helveticus*	*L. helveticus*	Kefir	221	ND	ND	ND	11.59	11.02	11.85
LUC-Lh030	*L. helveticus*	*L. helveticus*	Kefir	222	ND	ND	ND	11.35	11.68	11.02
LUC-Lh031	*L. helveticus*	*L. helveticus*	Buttermilk	223	ND	ND	ND	12.09	12.87	12.61

LUC-Lh032	*L. helveticus*	*L. helveticus*	Buttermilk	224	ND	ND	ND	14.03	14.80	14.62
LUC-Lh033	*L. helveticus*	*L. helveticus*	Buttermilk	225	ND	ND	ND	13.20	13.54	13.83
LUC-Lh034	*L. helveticus*	*L. helveticus*	Buttermilk	226	ND	ND	ND	10.88	10.53	10.64
LUC-Lh035	*L. helveticus*	*L. helveticus*	Buttermilk	227	ND	ND	ND	12.11	12.09	12.35
LUC-Lh036	*L. helveticus*	*L. helveticus*	Kombucha	228	ND	ND	ND	13.04	12.64	13.21
LUC-Lh037	*L. helveticus*	*L. helveticus*	Kombucha	229	ND	ND	ND	12.51	13.02	12.48
LUC-Lh038	*L. helveticus*	*L. helveticus*	Kombucha	230	ND	ND	ND	11.54	11.29	11.97
LUC-Lh039	*L. helveticus*	*L. helveticus*	Kombucha	231	ND	ND	ND	12.12	12.28	12.61
LUC-Lh040	*L. helveticus*	*L. helveticus*	Kimchi	232	ND	ND	ND	ND	ND	ND
LUC-Lh041	*L. helveticus*	*L. helveticus*	Kimchi	233	ND	ND	ND	ND	ND	ND
LUC-Lh042	*L. helveticus*	*L. helveticus*	Kimchi	234	ND	ND	ND	9.59	9.14	9.31
LUC-Lpl001	*Lactobacillus plantarum*	*Lactiplantibacillus plantarum*	Human saliva	235	ND	ND	ND	11.29	11.95	11.35
LUC-Lpl002	*L. plantarum*	*L. plantarum*	Human saliva	236	ND	ND	ND	9.53	9.49	9.30
LUC-Lpl003	*L. plantarum*	*L. plantarum*	Human saliva	237	ND	ND	ND	15.80	15.01	14.95
LUC-Lpl004	*L. plantarum*	*L. plantarum*	Human saliva	238	ND	ND	ND	ND	ND	ND
LUC-Lpl0005	*L. plantarum*	*L. plantarum*	Cabbage	239	ND	ND	ND	9.11	9.02	9.39
LUC-Lpl006	*L. plantarum*	*L. plantarum*	Cabbage	240	ND	ND	ND	ND	ND	ND
LUC-Lpl007	*L. plantarum*	*L. plantarum*	Cabbage	241	ND	ND	ND	14.66	15.03	14.25
LUC-Lpl008	*L. plantarum*	*L. plantarum*	Cabbage	242	ND	ND	ND	ND	ND	ND
LUC-Lpl009	*L. plantarum*	*L. plantarum*	Cabbage	243	ND	ND	ND	13.22	13.85	13.65
LUC-Lpl010	*L. plantarum*	*L. plantarum*	Spinach	244	ND	ND	ND	15.78	15.01	14.93
LUC-Lpl011	*L.s plantarum*	*L. plantarum*	Spinach	245	ND	ND	ND	ND	ND	ND
LUC-Lpl012	*L. plantarum*	*L. plantarum*	Spinach	246	ND	ND	ND	13.93	13.33	13.74
LUC-Lpl013	*L. plantarum*	*L. plantarum*	Spinach	247	ND	ND	ND	14.08	14.56	14.73
LUC-Lpl014	*L. plantarum*	*L. plantarum*	Spinach	248	ND	ND	ND	ND	ND	ND
LUC-Lpl015	*L. plantarum*	*L. plantarum*	Grass	249	ND	ND	ND	15.21	15.86	15.39
LUC-Lpl016	*L. plantarum*	*L. plantarum*	Grass	250	ND	ND	ND	14.9	14.91	15.38
LUC-Lpl017	*L. plantarum*	*L. plantarum*	Grass	251	ND	ND	ND	12.31	12.91	13.08
LUC-Lpl018	*L. plantarum*	*L. plantarum*	Healthy food	252	ND	ND	ND	12.38	12.37	13.55
LUC-Lpl019	*L. plantarum*	*L. plantarum*	Healthy food	253	ND	ND	ND	15.65	15.33	15.31
LUC-Lpl020	*L. plantarum*	*L. plantarum*	Healthy food	254	ND	ND	ND	13.28	13.93	13.62
LUC-Lpl021	*L. plantarum*	*L. plantarum*	Grass	255	ND	ND	ND	14.05	14.65	14.21
LUC-Lrh001	*Lactobacillus rhamnosus*	*Lacticaseibacillus rhamnosus*	Healthy food	256	ND	ND	ND	12.13	14.03	12.92

LUC-Lrh002	*L. rhamnosus*	*Lb. rhamnosus*	Healthy food	257	ND	ND	ND	13.05	13.91	13.46
LUC-Lrh003	*L. rhamnosus*	*Lb. rhamnosus*	Healthy food	258	ND	ND	ND	12.11	12.99	13.01
LUC-Lrh004	*L. rhamnosus*	*Lb. rhamnosus*	Healthy food	259	ND	ND	ND	14.46	14.93	15.02
LUC-Lrh005	*L. rhamnosus*	*Lb. rhamnosus*	Healthy food	260	ND	ND	ND	16.98	16.16	16.37
LUC-Lrh006	*L. rhamnosus*	*Lb. rhamnosus*	Fermented milk	261	ND	ND	ND	15.01	16.54	15.47
LUC-Lrh007	*L. rhamnosus*	*Lb. rhamnosus*	Fermented milk	262	ND	ND	ND	17.87	16.96	17.35
LUC-Lrh008	*L. rhamnosus*	*Lb. rhamnosus*	Healthy food	263	ND	ND	ND	18.02	18.45	18.95
LUC-Lrh009	*L. rhamnosus*	*Lb. rhamnosus*	Healthy food	264	ND	ND	ND	20.11	20.22	20.85
LUC-Lrh010	*L. rhamnosus*	*Lb. rhamnosus*	Female urinary	265	ND	ND	ND	19.02	19.81	19.34
LUC-Lrh012	*L. rhamnosus*	*Lb. rhamnosus*	Female urinary	266	ND	ND	ND	16.79	15.94	16.88
LUC-Lrh013	*L. rhamnosus*	*Lb. rhamnosus*	Female urinary	267	ND	ND	ND	20.35	20.48	20.19
LUC-Lre001	*Lactobacillus reuteri*	*Limosilactobacillus reuteri*	Human intestine	268	ND	ND	ND	21.20	21.23	21.64
LUC-Lre002	*L. reuteri*	*L. reuteri*	Human intestine	269	ND	ND	ND	18.51	18.92	18.43
LUC-Lre003	*L. reuteri*	*L. reuteri*	Human intestine	270	ND	ND	ND	15.90	16.85	16.25
LUC-Lre004	*L. reuteri*	*L. reuteri*	Human intestine	271	ND	ND	ND	29.81	29.91	28.81
LUC-Lre005	*L. reuteri*	*L. reuteri*	Human intestine	272	ND	ND	ND	30.11	30.06	30.62
LUC-Lre006	*L. reuteri*	*L. reuteri*	Cow intestine	273	ND	ND	ND	25.40	25.02	25.97
LUC-Lre007	*L. reuteri*	*L. reuteri*	Cow intestine	274	ND	ND	ND	26.21	25.92	25.66
LUC-Lre008	*L. reuteri*	*L. reuteri*	Cow intestine	275	ND	ND	ND	20.21	20.97	20.02
LUC-Lre009	*L. reuteri*	*L. reuteri*	Cow intestine	276	ND	ND	ND	19.25	19.32	19.85
LUC-Lre010	*L. reuteri*	*L. reuteri*	Cow intestine	277	ND	ND	ND	ND	ND	ND
LUC-Lre011	*L. reuteri*	*L. reuteri*	Healthy food	278	ND	ND	ND	9.18	9.55	9.38
LUC-Lre012	*L. reuteri*	*L. reuteri*	Healthy food	279	ND	ND	ND	ND	ND	ND
LUC-Lre013	*L. reuteri*	*L. reuteri*	Healthy food	280	ND	ND	ND	9.65	9.88	10.02
LUC-Lre014	*L. reuteri*	*L. reuteri*	Healthy food	281	ND	ND	ND	8.75	8.92	9.04
LUC-Lre015	*L. reuteri*	*L. reuteri*	Healthy food	282	ND	ND	ND	12.11	12.38	12.99
LUC-Lre016	*L. reuteri*	*L. reuteri*	Healthy food	283	ND	ND	ND	9.59	9.01	9.25
LUC-Lre017	*L. reuteri*	*L. reuteri*	Healthy food	284	ND	ND	ND	ND	ND	ND
LUC-Ls001	*Lactobacillus salivarius*	*Ligilactobacillus salivarius*	Human intestine	285	ND	ND	ND	30.53	30.34	30.02
LUC-Ls002	*L. salivarius*	*Llb. salivarius*	Human intestine	286	ND	ND	ND	ND	ND	ND
LUC-Ls003	*L. salivarius*	*Llb. salivarius*	Human intestine	287	ND	ND	ND	12.65	12.05	12.34
LUC-Ls004	*L. salivarius*	*Llb. salivarius*	Human intestine	288	ND	ND	ND	15.68	15.02	15.33
LUC-Ls005	*L. salivarius*	*Llb. salivarius*	Human intestine	289	ND	ND	ND	15.07	14.56	14.94

LUC-Ls006	*L. salivarius*	*Llb. salivarius*	Human intestine	290	ND	ND	ND	14.05	14.39	14.58
LUC-Ls007	*L. salivarius*	*Llb. salivarius*	Human saliva	291	ND	ND	ND	8.74	8.29	8.37
LUC-Ls008	*L. salivarius*	*Llb. salivarius*	Human saliva	292	ND	ND	ND	12.01	12.56	12.69
LUC-Ls009	*L. salivarius*	*Llb. salivarius*	Human saliva	293	ND	ND	ND	14.41	14.30	14.72
LUC-Ls010	*L. salivarius*	*Llb. salivarius*	Human saliva	294	ND	ND	ND	ND	ND	ND
LUC-Ls011	*L. salivarius*	*Llb. salivarius*	Human saliva	295	ND	ND	ND	29.56	29.43	30.91
LUC-Ls012	*L. salivarius*	*Llb. salivarius*	Human saliva	296	ND	ND	ND	24.30	24.31	23.96
LUC-Ls013	*L. salivarius*	*Llb. salivarius*	Human saliva	297	ND	ND	ND	23.22	24.57	24.12
LUC-Ls014	*L. salivarius*	*Llb. salivarius*	Human saliva	298	ND	ND	ND	ND	ND	ND
LUC-Ls015	*L. salivarius*	*Llb. salivarius*	Healthy food	299	ND	ND	ND	ND	ND	ND
LUC-Ls016	*L. salivarius*	*Llb. salivarius*	Healthy food	300	ND	ND	ND	ND	ND	ND
LUC-Ls017	*L. salivarius*	*Llb. salivarius*	Healthy food	301	ND	ND	ND	20.28	19.54	21.16
LUC-Ls018	*L. salivarius*	*Llb. salivarius*	Healthy food	302	ND	ND	ND	21.20	21.87	23.18
LUC-Ls019	*L. salivarius*	*Llb. salivarius*	Healthy food	303	ND	ND	ND	23.72	24.17	24.02
LUC-Ls020	*L. salivarius*	*Llb. salivarius*	Healthy food	304	ND	ND	ND	19.22	20.17	21.17
LUC-Ls021	*L. salivarius*	*Llb. salivarius*	Healthy food	305	ND	ND	ND	20.22	20.97	21.02
LUC-Ls022	*L. salivarius*	*Llb. salivarius*	Healthy food	306	ND	ND	ND	21.29	22.87	23.23
LUC-Ls023	*L. salivarius*	*Llb. salivarius*	Healthy food	307	ND	ND	ND	24.22	25.57	24.92
LUC-Ls024	*L. salivarius*	*Llb. salivarius*	Healthy food	308	ND	ND	ND	ND	ND	ND
LUC-Ls025	*L. salivarius*	*Llb. salivarius*	Human intestine	309	ND	ND	ND	ND	ND	ND
LUC-Ls026	*L. salivarius*	*Llb. salivarius*	Human intestine	310	ND	ND	ND	ND	ND	ND
LUC-Ls027	*L. salivarius*	*Llb. salivarius*	Human intestine	311	ND	ND	ND	18.72	19.27	18.07
LUC-Ls028	*L. salivarius*	*Llb. salivarius*	Human intestine	312	ND	ND	ND	19.44	21.37	21.57
LUC-Ls029	*L. salivarius*	*Llb. salivarius*	Human intestine	313	ND	ND	ND	17.29	17.17	17.67
LUC-Ls030	*L. salivarius*	*Llb. salivarius*	Human intestine	314	ND	ND	ND	21.52	21.17	22.36
LUC-Ls031	*L. salivarius*	*Llb. salivarius*	Human intestine	315	ND	ND	ND	18.29	19.44	19.67
LUC-Ls032	*L. salivarius*	*Llb. salivarius*	Healthy food	316	ND	ND	ND	17.72	17.25	18.31
LUC-Ls033	*L. salivarius*	*Llb. salivarius*	Healthy food	317	ND	ND	ND	19.02	20.11	21.99
LUC-Ls034	*L. salivarius*	*Llb. salivarius*	Healthy food	318	ND	ND	ND	ND	ND	ND
LUC-Ls035	*L. salivarius*	*Llb. salivarius*	Healthy food	319	ND	ND	ND	10.24	10.36	10.61
LUC-Ls036	*L. salivarius*	*Llb. salivarius*	Healthy food	320	ND	ND	ND	ND	ND	ND
LUC-Ls037	*L. salivarius*	*Llb. salivarius*	Healthy food	321	ND	ND	ND	25.02	26.11	25.93
LUC-Ls038	*L. salivarius*	*Llb. salivarius*	Healthy food	322	ND	ND	ND	24.42	23.81	24.499
LUC-Ls039	*L. salivarius*	*Llb. salivarius*	Healthy food	323	ND	ND	ND	19.02	20.11	21.99

LUC-Ls040	*L. salivarius*	*Llb. salivarius*	Healthy food	324	ND	ND	ND	ND	ND	ND
LUC-Ls041	*L. salivarius*	*Llb. salivarius*	Healthy food	325	ND	ND	ND	ND	ND	ND
LUC-Ls042	*L. salivarius*	*Llb. salivarius*	Human teeth	326	ND	ND	ND	28.60	28.32	28.11
LUC-Ls043	*L. salivarius*	*Llb. salivarius*	Human teeth	327	ND	ND	ND	28.03	28.12	28.34
LUC-Ls044	*L. salivarius*	*Llb. salivarius*	Human teeth	328	ND	ND	ND	27.56	27.3	27.64
LUC-Ls045	*L. salivarius*	*Llb. salivarius*	Human teeth	329	ND	ND	ND	18.32	18.21	18.62
LUC-Ls046	*L. salivarius*	*Llb. salivarius*	Human teeth	330	ND	ND	ND	21.03	21.44	21.09
LUC-Ls047	*L. salivarius*	*Llb. salivarius*	Human teeth	331	ND	ND	ND	24.06	24.99	25
LUC-Ls048	*L. salivarius*	*Llb. salivarius*	Human teeth	332	ND	ND	ND	30.11	30.65	30.12
LUC-Ls049	*L. salivarius*	*Llb. salivarius*	Human teeth	333	ND	ND	ND	19.25	19.09	19.83
LUC-Ls050	*L. salivarius*	*Llb. salivarius*	Human stomach	334	ND	ND	ND	ND	ND	ND
LUC-Ls051	*L. salivarius*	*Llb. salivarius*	Human stomach	335	ND	ND	ND	27.07	26.99	26.62
LUC-Ls052	*L. salivarius*	*Llb. salivarius*	Human stomach	336	ND	ND	ND	25.56	25.92	25.6
LUC-Ls053	*L. salivarius*	*Llb. salivarius*	Human stomach	337	ND	ND	ND	23.07	23.84	23.5
LUC-Ls054	*L. salivarius*	*Llb. salivarius*	Human stomach	338	ND	ND	ND	ND	ND	ND
LUC-Ls055	*L. salivarius*	*Llb. salivarius*	Human stomach	339	ND	ND	ND	26.08	26.94	26.23
LUC-Ls056	*L. salivarius*	*Llb. salivarius*	Human stomach	340	ND	ND	ND	ND	ND	ND
LUC-Ls057	*L. salivarius*	*Llb. salivarius*	Human stomach	341	ND	ND	ND	28.16	28.95	28.00
LUC-Ls058	*L. salivarius*	*Llb. salivarius*	Human stomach	342	ND	ND	ND	ND	ND	ND
LUC-Ls059	*L. salivarius*	*Llb. salivarius*	Healthy food	343	ND	ND	ND	22.16	22.29	22.41
LUC-Ls060	*L. salivarius*	*Llb. salivarius*	Healthy food	344	ND	ND	ND	22.09	22.39	22.80
LUC-Ls061	*L. salivarius*	*Llb. salivarius*	Healthy food	345	ND	ND	ND	27.26	27.89	26.99
LUC-Ls062	*L. salivarius*	*Llb. salivarius*	Healthy food	346	ND	ND	ND	23.09	23.77	23.25
LUC-Ls063	*L. salivarius*	*Llb. salivarius*	Healthy food	347	ND	ND	ND	ND	ND	ND
LUC-Ls064	*L. salivarius*	*Llb. salivarius*	Healthy food	348	ND	ND	ND	ND	ND	ND
LUC-Ls065	*L. salivarius*	*Llb. salivarius*	Healthy food	349	ND	ND	ND	29.16	29.53	29.06
LUC-Ls066	*L. salivarius*	*Llb. salivarius*	Human teeth	350	ND	ND	ND	28.01	27.97	28.07
LUC-Ls067	*L. salivarius*	*Llb. salivarius*	Human teeth	351	ND	ND	ND	ND	ND	ND
LUC-Ls068	*L. salivarius*	*Llb. salivarius*	Human teeth	352	ND	ND	ND	20.76	20.55	20.83
LUC-Ls069	*L. salivarius*	*Llb. salivarius*	Human teeth	353	ND	ND	ND	24.36	24.69	24.09
LUC-Ls070	*L. salivarius*	*Llb. salivarius*	Human teeth	354	ND	ND	ND	21.86	21.90	21.82
LUC-Ls071	*L. salivarius*	*Llb. salivarius*	Human teeth	355	ND	ND	ND	21.06	21.29	21.37
LUC-Ls072	*L. salivarius*	*Llb. salivarius*	Human stomach	356	ND	ND	ND	ND	ND	ND
LUC-Ls073	*L. salivarius*	*Llb. salivarius*	Human stomach	357	ND	ND	ND	ND	ND	ND

LUC-Ls074	*L. salivarius*	*Llb. salivarius*	Human stomach	358	ND	ND	ND	23.09	23.91	23.55
LUC-Ls075	*L. salivarius*	*Llb. salivarius*	Human stomach	359	ND	ND	ND	ND	ND	ND
LUC-Ls076	*L. salivarius*	*Llb. salivarius*	Human stomach	360	ND	ND	ND	25.46	25.99	25.37
LUC-Ls077	*L. salivarius*	*Llb. salivarius*	Human stomach	361	ND	ND	ND	22.09	23.69	23.48
LUC-Ls078	*L. salivarius*	*Llb. salivarius*	Human stomach	362	ND	ND	ND	ND	ND	ND
LUC-Ff001	*Fructobacillus fructosus*	*Fructobacillus fructosus*	Honeybees	363	ND	ND	ND	ND	ND	ND
LUC-Ff002	*F. fructosus*	*F. fructosus*	Honeybees	364	ND	ND	ND	11.36	11.98	11.05
LUC-Ff003	*F. fructosus*	*F. fructosus*	Honeybees	365	ND	ND	ND	10.98	10.01	10.56
LUC-Ff004	*F. fructosus*	*F. fructosus*	Honeybees	366	ND	ND	ND	13.25	13.13	13.1
LUC-Ff005	*F. fructosus*	*F. fructosus*	Honeybees	367	ND	ND	ND	ND	ND	ND
LUC-Ff006	*F. fructosus*	*F. fructosus*	Honeybees	368	ND	ND	ND	19.25	19.09	19.83
LUC-Ff007	*F. fructosus*	*F. fructosus*	Honeybees	369	ND	ND	ND	ND	ND	ND
LUC-Ff008	*F. fructosus*	*F. fructosus*	Honeybees	370	ND	ND	ND	19.25	19.09	19.83
LUC-Ff009	*F. fructosus*	*F. fructosus*	Fructose factory	371	ND	ND	ND	19.25	19.09	19.83
LUC-Ff010	*F. fructosus*	*F. fructosus*	Fructose factory	372	ND	ND	ND	19.25	19.09	19.83
LUC-Ff011	*F. fructosus*	*F. fructosus*	Fructose factory	373	ND	ND	ND	ND	ND	ND
LUC-Ff012	*F. fructosus*	*F. fructosus*	Fructose factory	374	ND	ND	ND	ND	ND	ND
LUC-Ff013	*F. fructosus*	*F. fructosus*	Mango flower	375	ND	ND	ND	19.25	19.09	19.83
LUC-Ff014	*F. fructosus*	*F. fructosus*	Mango flower	376	ND	ND	ND	19.25	19.09	19.83
LUC-Ff015	*F. fructosus*	*F. fructosus*	Mango flower	377	ND	ND	ND	19.25	19.09	19.83
LUC-Ff016	*F. fructosus*	*F. fructosus*	Mango flower	378	ND	ND	ND	19.25	19.09	19.83
LUC-Ff017	*F. fructosus*	*F. fructosus*	Fructose factory	379	ND	ND	ND	ND	ND	ND
LUC-Ff018	*F. fructosus*	*F. fructosus*	Fructose factory	380	ND	ND	ND	ND	ND	ND
LUC-Ff019	*F. fructosus*	*F. fructosus*	Banana	381	ND	ND	ND	ND	ND	ND
LUC-Ff020	*F. fructosus*	*F. fructosus*	Banana	382	ND	ND	ND	19.25	19.09	19.83
LUC-Ff021	*F. fructosus*	*F. fructosus*	Banana	383	ND	ND	ND	ND	ND	ND
LUC-Ff022	*F. fructosus*	*F. fructosus*	Banana	384	ND	ND	ND	ND	ND	ND
LUC-Ff023	*F. fructosus*	*F. fructosus*	Banana	385	ND	ND	ND	9.12	9.06	9.31
LUC-Ff024	*F. fructosus*	*F. fructosus*	Longan Flower	386	ND	ND	ND	ND	ND	ND
LUC-Ff025	*F. fructosus*	*F. fructosus*	Longan Flower	387	ND	ND	ND	ND	ND	ND
LUC-Ff026	*F. fructosus*	*F. fructosus*	Longan Flower	388	ND	ND	ND	ND	ND	ND
LUC-Ff027	*F. fructosus*	*F. fructosus*	Longan Flower	389	ND	ND	ND	11.51	10.93	10.52
LUC-St001	*Streptococcus thermophilus*	*Streptococcus thermophilus*	Fermented milk	390	ND	ND	ND	ND	ND	ND

LUC-St002	*S. thermophilus*	*S. thermophilus*	Fermented milk	391	ND	ND	ND	13.02	13.46	13.98
LUC-St003	*S. thermophilus*	*S. thermophilus*	Fermented milk	392	ND	ND	ND	10.69	10.86	11.25
LUC-St004	*S. thermophilus*	*S. thermophilus*	Fermented milk	393	ND	ND	ND	ND	ND	ND
LUC-St005	*S. thermophilus*	*S. thermophilus*	Fermented milk	394	ND	ND	ND	ND	ND	ND
LUC-St006	*S. thermophilus*	*S. thermophilus*	Fermented milk	395	ND	ND	ND	ND	ND	ND
LUC-St007	*S. thermophilus*	*S. thermophilus*	Fermented milk	396	ND	ND	ND	ND	ND	ND
LUC-St008	*S. thermophilus*	*S. thermophilus*	Fermented milk	397	ND	ND	ND	10.21	10.63	10.67
LUC-St009	*S. thermophilus*	*S. thermophilus*	Fermented milk	398	ND	ND	ND	13.42	13.95	13.35
LUC-St010	*S. thermophilus*	*S. thermophilus*	Fermented milk	399	ND	ND	ND	ND	ND	ND
LUC-St011	*S. thermophilus*	*S. thermophilus*	Fermented milk	400	ND	ND	ND	9.50	9.43	9.12
LUC-St012	*S. thermophilus*	*S. thermophilus*	Yogurt	401	ND	ND	ND	ND	ND	ND
LUC-St013	*S. thermophilus*	*S. thermophilus*	Yogurt	402	ND	ND	ND	10.25	10.39	10.87
LUC-St014	*S. thermophilus*	*S. thermophilus*	Yogurt	403	ND	ND	ND	ND	ND	ND
LUC-St015	*S. thermophilus*	*S. thermophilus*	Yogurt	404	ND	ND	ND	12.54	13.01	13.87
LUC-St016	*S. thermophilus*	*S. thermophilus*	Yogurt	405	ND	ND	ND	ND	ND	ND
LUC-St017	*S. thermophilus*	*S. thermophilus*	Yogurt	406	ND	ND	ND	10.15	10.29	10.33
LUC-St018	*S. thermophilus*	*S. thermophilus*	Yogurt	407	ND	ND	ND	12.02	12.34	12.51
LUC-St019	*S. thermophilus*	*S. thermophilus*	Cheese	408	ND	ND	ND	ND	ND	ND
LUC-St020	*S. thermophilus*	*S. thermophilus*	Cheese	409	ND	ND	ND	10.39	10.11	10.64
LUC-St021	*S. thermophilus*	*S. thermophilus*	Cheese	410	ND	ND	ND	ND	ND	ND
LUC-St022	*S. thermophilus*	*S. thermophilus*	Cheese	411	ND	ND	ND	11.23	11.95	11.34
LUC-St023	*S. thermophilus*	*S. thermophilus*	Cheese	412	ND	ND	ND	ND	ND	ND
LUC-St024	*S. thermophilus*	*S. thermophilus*	Cheese	413	ND	ND	ND	12.60	11.72	11.01
LUC-St025	*S. thermophilus*	*S. thermophilus*	Cheese	414	ND	ND	ND	11.00	12.02	12.4
LUC-St026	*S. thermophilus*	*S. thermophilus*	Cheese	415	ND	ND	ND	9.83	9.99	9.01
LUC-St027	*S. thermophilus*	*S. thermophilus*	Cheese	416	ND	ND	ND	ND	ND	ND
LUC-St028	*S. thermophilus*	*S. thermophilus*	Yogurt	417	ND	ND	ND	ND	ND	ND
LUC-St029	*S. thermophilus*	*S. thermophilus*	Yogurt	418	ND	ND	ND	9.44	9.01	9.99
LUC-St030	*S. thermophilus*	*S. thermophilus*	Yogurt	419	ND	ND	ND	ND	ND	ND
LUC-St031	*S. thermophilus*	*S. thermophilus*	Yogurt	420	ND	ND	ND	16.19	15.08	15.68
LUC-St032	*S. thermophilus*	*S. thermophilus*	Yogurt	421	ND	ND	ND	10.98	10.55	10.37
LUC-St033	*S. thermophilus*	*S. thermophilus*	Fermented milk	422	ND	ND	ND	9.15	9.68	9.35
LUC-St034	*S. thermophilus*	*S. thermophilus*	Fermented milk	423	ND	ND	ND	12.21	12.93	12.55
LUC-St035	*S. thermophilus*	*S. thermophilus*	Fermented milk	424	ND	ND	ND	11.55	11.69	11.37

LUC-St036	*S. thermophilus*	*S. thermophilus*	Fermented milk	425	ND	ND	ND	13.24	13.32	13.1
LUC-St037	*S. thermophilus*	*S. thermophilus*	Fermented milk	426	ND	ND	ND	9.26	9.27	9.64
LUC-St038	*S. thermophilus*	*S. thermophilus*	Fermented milk	427	ND	ND	ND	9.34	9.12	9.5
LUC-Bl001	*Bifidobacterium lactis*	*Bifidobacterium lactis*	Baby feces	428	ND	ND	ND	ND	ND	ND
LUC-Bl002	*B. lactis*	*B. lactis*	Baby feces	429	ND	ND	ND	12.50	11.90	11.48
LUC-Bl003	*B. lactis*	*B. lactis*	Baby feces	430	ND	ND	ND	10.90	10.62	10.67
LUC-Bl004	*B. lactis*	*B. lactis*	Baby feces	431	ND	ND	ND	ND	ND	ND
LUC-Bl005	*B. lactis*	*B. lactis*	Baby feces	432	ND	ND	ND	ND	ND	ND
LUC-Bl006	*B. lactis*	*B. lactis*	Baby feces	433	ND	ND	ND	10.39	10.52	10.31
LUC-Bl007	*B. lactis*	*B. lactis*	Baby feces	434	ND	ND	ND	ND	ND	ND
LUC-Bl008	*B. lactis*	*B. lactis*	Baby feces	435	ND	ND	ND	12.21	12.63	12.84
LUC-Bl009	*B. lactis*	*B. lactis*	Human intestine	436	ND	ND	ND	ND	ND	ND
LUC-Bl010	*B. lactis*	*B. lactis*	Human intestine	437	ND	ND	ND	ND	ND	ND
LUC-Bl011	*B. lactis*	*B. lactis*	Human intestine	438	ND	ND	ND	10.98	10.12	10.6
LUC-Bl012	*B. lactis*	*B. lactis*	Human intestine	439	ND	ND	ND	12.32	12.08	12.61
LUC-Bl013	*B. lactis*	*B. lactis*	Human intestine	440	ND	ND	ND	10.69	10.23	10.87
LUC-Bl014	*B. lactis*	*B. lactis*	Human intestine	441	ND	ND	ND	ND	ND	ND
LUC-Bl015	*B. lactis*	*B. lactis*	Human intestine	442	ND	ND	ND	9.59	9.99	9.01
LUC-Bl016	*B. lactis*	*B. lactis*	Healthy food	443	ND	ND	ND	ND	ND	ND
LUC-Bl017	*B. lactis*	*B. lactis*	Healthy food	444	ND	ND	ND	15.82	15.98	14.95
LUC-Bl018	*B. lactis*	*B. lactis*	Healthy food	445	ND	ND	ND	ND	ND	ND
LUC-Bl019	*B. lactis*	*B. lactis*	Healthy food	446	ND	ND	ND	ND	ND	ND
LUC-Bl020	*B. lactis*	*B. lactis*	Healthy food	447	ND	ND	ND	10.21	10.68	10.34
LUC-Bl021	*B. lactis*	*B. lactis*	Healthy food	448	ND	ND	ND	ND	ND	ND
LUC-Bl022	*B. lactis*	*B. lactis*	Healthy food	449	ND	ND	ND	8.59	8.95	8.68
LUC-Bl023	*B. lactis*	*B. lactis*	Healthy food	450	ND	ND	ND	ND	ND	ND

[a] p<0.01.414

[b] p<0.01.

Table S2. The factors and levels per factor of the first experiments for *Lb. paracasei* 111 and *Lb. paracasei* 141.

**Levels**	**Factors**						
	(A) [b] Medium	(B) Growth	(C) Culturing	(D) Adjusted	(E) Amount	(F) Feed	(G) Culturing

		factors	temp.	pH	of starter	speed	time
1 [a]	M [c] 10.0% + G [d] 2.0%	2.0%	36	5.8	3.0%	10%/hour, for 10 hour	12 hours
2	M 8.0% + G3.0%	1.5%	39	6.3	5.0%	10%/hour, for 8 hour	16 hours
3	M 6.0% + G4.0%	1.0%	42	6.8	7.0%	10%/hour, for 6 hour	-

[a] 1, 2, 3 were level serial code number.

[b] (A), (B), ….., (G) were codenames of factors.

[c] Symbol M was skim milk.

[d] Symbol G was glucose.

Table S3. The design of experiments and the results of the first experiment of optimal culture media and conditions for *Lb. paracasei* 111 (unit: 1.0×10^8 cfu / ml).

**Experiment codes**	**Factors**								**Results of *Lb. paracasei* 111 count**	
	(G) [a] Culturing time	(A) Medium	(B) Growth factors	(C) Culturing temp.	(D) Adjusted pH	(E) Amount of starter	(F) Feed speed	*error*		
1	1 [b]	1	1	1	1	1	1	1	34	39
2	1	1	2	2	2	2	2	2	38	33
3	1	1	3	3	3	3	3	3	33	36
4	1	2	1	1	2	2	3	3	36	40
5	1	2	2	2	3	3	1	1	42	38
6	1	2	3	3	1	1	2	2	31	24
7	1	3	1	2	1	3	2	3	32	37
8	1	3	2	3	2	1	3	1	26	20
9	1	3	3	1	3	2	1	2	27	19
10	2	1	1	3	3	2	2	1	39	34
11	2	1	2	1	1	3	3	2	33	39
12	2	1	3	2	2	1	1	3	35	41
13	2	2	1	2	3	1	3	2	39	33
14	2	2	2	3	1	2	1	3	47	40
15	2	2	3	1	2	3	2	1	32	24

16	2	3	1	3	2	3	1	2	34	39
17	2	3	2	1	3	1	2	3	32	26
18	2	3	3	2	1	2	3	1	28	20
L_1 [d]	32.50	36.17	36.33	31.75	33.67	31.67	36.25	-	-	-
L_2	34.17	35.50	34.50	34.67	33.17	33.42	31.83	-	-	-
L_3	-	28.33	29.17	33.58	33.17	34.92	31.92	-	-	-
R [e]	1.67	7.83	7.17	2.92	0.50	3.25	4.42	-	-	-
O [f]	2	1	1	2	1	3	1	-	-	-
mean	33.33	33.33	33.33	33.33	33.33	33.33	33.33	-	33.33	
SD	-	-	-	-	-	-	-	-	6.69	

[a] (A), (B), ….., (G) were codenames of factors. Each column was configured with one factor.

[b] 1, 2, 3 were level serial number.

[c] Level value was listed in Table S1.

[d] L_1 was the mean (unit: 1.0×10^8 cfu / ml) of all result values of level 1 of some factor, for example, L_1 of factor A = (34+39+38+33+33+36+39+34+33+39+35+41)/12 = 36.17, L_2 was the mean of all resulted values of level 2 of some factor, and L_3 was the mean of all result values of level 3 of some factor.

[e] R was the range of L_1, L_2, L_3 of some factor.

[f] O was the optimal level of some factor; SD was the standard deviation of all result data.

Table S4. The factors and levels per factor of the second experiments for *Lb. paracasei* 111 and *Lb. paracasei* 141.

Levels	Factors			
	(A) [b] Medium	(B) Growth factors	(C) Culturing temp.	(E) Amount of starter
1 [a]	M [c] 7.0% + G [d] 3.5%	2.0%	37	10%/hour for 10 hours
2	M 8.0% + G 3.0%	2.5%	39	9%/hour for 11.2 hours
3	M 9.0% + G 2.5%	3.0%	41	8%/hour for 12.5 hours

[a] 1, 2, 3 were level serial code number.

[b] (A), (B), ….., (G) were codenames of factors.

[c] Symbol M was skim milk.

[d] Symbol G was glucose.

Table S5. The design of experiment and the results of the second experiment of optimal culture media and culture conditions for *Lb. paracasei* 111 (unit: 1.0 × 10^8 cfu / ml).

**No**	**1**	**2**	**3**	**4**	**Results of *Lb. paracasei* 111 count**	
Factor	(A) Medium	(B) Growth factors	(C) Culturing temp.	(D) Feed speed		
1	1	1	1	1	30	33
2	1	2	2	2	41	45
3	1	3	3	3	35	40
4	2	1	2	3	40	44
5	2	2	3	1	39	35
6	2	3	1	2	45	49
7	3	1	3	2	42	37
8	3	2	1	3	39	36
9	3	3	2	1	34	38
L_1	37.33	37.67	38.67	34.83	-	-
L_2	42.00	39.17	40.33	43.17	-	-
L_3	37.67	40.17	38.00	39.00	-	-
R	4.67	2.50	2.33	8.33	-	-
O	2	3	2	2	-	-
mean	39.00	39.00	39.00	39.00	39.00	
SD	-	-	-	-	4.85	

Note: In Table S4, the meanings of M, G, L_1, L_2, L_3, R, O, and SD were the same as the description of note in S2 Table.

Table S6. The design of experiment and the results of the first experiment of optimal culture media and culture conditions for *Lb. paracasei* 141 (unit: 1.0 × 10^8 cfu / ml).

Experiment codes	Factors								Results of *Lb. paracasei* 141 count	
	(G) [a] Culturing time	(A) Medium	(B) Growth factors	(C) Culturing temp.	(D) Adjusted pH	(E) Amount of starter	(F) Feed speed	error		
1	1	1	1	1	1	1	1	1	29	34
2	1	1	2	2	2	2	2	2	37	31
3	1	1	3	3	3	3	3	3	28	23
4	1	2	1	1	2	2	3	3	31	37
5	1	2	2	2	3	3	1	1	29	21
6	1	2	3	3	1	1	2	2	24	17
7	1	3	1	2	1	3	2	3	20	28
8	1	3	2	3	2	1	3	1	23	29
9	1	3	3	1	3	2	1	2	29	24
10	2	1	1	3	3	2	2	1	34	25
11	2	1	2	1	1	3	3	2	29	36
12	2	1	3	2	2	1	1	3	28	34
13	2	2	1	2	3	1	3	2	26	20
14	2	2	2	3	1	2	1	3	34	27
15	2	2	3	1	2	3	2	1	29	24
16	2	3	1	3	2	3	1	2	21	28
17	2	3	2	1	3	1	2	3	26	19
18	2	3	3	2	1	2	3	1	21	27
L_1	27.44	30.67	27.75	28.92	27.17	25.75	28.17	-	-	-
L_2	27.11	26.58	28.42	26.83	29.33	29.75	26.17	-	-	-
L_3	-	24.58	25.67	26.08	25.33	26.33	27.50	-	-	-
R	0.33	6.08	2.75	2.83	4.00	4.00	2.00	-	-	-
O	1	1	2	1	2	2	1	-	-	-
mean	27.28	27.28	27.28	27.28	27.28	27.28	27.28	-	27.28	
SD	-	-	-	-	-	-	-	-	5.26	

Note: In Table S5, the meanings of L_1, L_2, L_3, R, O, and SD were the same as the description of note in S2 Table.

Table S7. The design of experiment and the results of the second experiment of optimal culture media and culture conditions for *Lb. paracasei* 141 (unit: 1.0 × 10^8 cfu / ml).

Factor	**1** (A) Medium	**2** (B) Growth factors	**3** (C) Culturing temp.	**4** (D) Feed speed	**Results** ***Lb. paracasei*** **141 count**	
1	1	1	1	1	23	27
2	1	2	2	2	34	37
3	1	3	3	3	27	31
4	2	1	2	3	34	37
5	2	2	3	1	34	29
6	2	3	1	2	39	43
7	3	1	3	2	35	29
8	3	2	1	3	34	33
9	3	3	2	1	27	31
L_1	28.00	30.67	32.00	28.00	-	-
L_2	35.67	34.00	31.67	36.00	-	-
L_3	32.00	31.00	32.00	31.67	-	-
R	7.67	3.33	0.33	8.00	-	-
O	2	2	3	2	-	-
mean	32.44	32.44	32.44	32.44	32.44	
SD	-	-	-	-	4.96	

Note: In Table S6, the meanings of L_1, L_2, L_3, R, O, and SD were the same as the description of note in S2 Table.

Table S8. The predicted product self-life at 25°C and the actual measured quality value at this shelf-life.
